# Efficacy of non-invasive brain stimulation on cognitive functioning in brain disorders: a meta-analysis

**DOI:** 10.1017/S0033291720003670

**Published:** 2020-11

**Authors:** Marieke J. Begemann, Bodyl A. Brand, Branislava Ćurčić-Blake, André Aleman, Iris E. Sommer

**Affiliations:** Department of Biomedical Sciences of Cells & Systems, Section Cognitive Neurosciences, University Medical Center Groningen, University of Groningen, Groningen, The Netherlands

**Keywords:** Brain disorder, cognitive dysfunction, non-invasive brain stimulation, prefrontal cortex, repetitive transcranial magnetic stimulation, transcranial direct current stimulation

## Abstract

**Background:**

Cognition is commonly affected in brain disorders. Non-invasive brain stimulation (NIBS) may have procognitive effects, with high tolerability. This meta-analysis evaluates the efficacy of transcranial magnetic stimulation (TMS) and transcranial Direct Current Stimulation (tDCS) in improving cognition, in schizophrenia, depression, dementia, Parkinson's disease, stroke, traumatic brain injury, and multiple sclerosis.

**Methods:**

A PRISMA systematic search was conducted for randomized controlled trials. Hedges' *g* was used to quantify effect sizes (ES) for changes in cognition after TMS/tDCS *v.* sham. As different cognitive functions may have unequal susceptibility to TMS/tDCS, we separately evaluated the effects on: attention/vigilance, working memory, executive functioning, processing speed, verbal fluency, verbal learning, and social cognition.

**Results:**

We included 82 studies (*n* = 2784). For working memory, both TMS (ES = 0.17, *p* = 0.015) and tDCS (ES = 0.17, *p* = 0.021) showed small but significant effects. Age positively moderated the effect of TMS. TDCS was superior to sham for attention/vigilance (ES = 0.20, *p* = 0.020). These significant effects did not differ across the type of brain disorder. Results were not significant for the other five cognitive domains.

**Conclusions:**

Our results revealed that both TMS and tDCS elicit a small trans-diagnostic effect on working memory, tDCS also improved attention/vigilance across diagnoses. Effects on the other domains were not significant. Observed ES were small, yet even slight cognitive improvements may facilitate daily functioning. While NIBS can be a well-tolerated treatment, its effects appear domain specific and should be applied only for realistic indications (i.e. to induce a small improvement in working memory or attention).

## Introduction

Cognitive functioning is affected in many brain disorders (Robbins, 2011). The observed impairment can be profound, as in Alzheimer's disease or Parkinson's disease (PD), or relatively mild, as in depression (Brown & Marsden, 1990; Caviness et al., 2007; Douglas et al., 2018; Petersen et al., 2001). Other brain disorders, such as multiple sclerosis (MS), schizophrenia or traumatic brain injury (TBI) can co-occur with more varying levels of cognitive performance, with either mild or severe cognitive dysfunction (Chiaravalloti & DeLuca, 2008; Heinrichs & Zakzanis, 1998; Sun, Tan, & Yu, 2014; Walker & Tesco, 2013). Impairments may affect multiple cognitive domains, including information processing, working memory, executive functioning and attention (Caviness et al., 2007; Higuchi et al., 2017; Maloni, 2018). These disturbances can profoundly impact daily functioning and quality of life (Papakostas et al., 2004; Schrag, Jahanshahi, & Quinn, 2000). Cognition determines, to a considerable extent, one's social and professional success and the ability to live independently (Audet, Hebert, Dubois, Rochette, & Mercier, 2007; Benedict & Zivadinov, 2006; Green, Kern, Braff, & Mintz, 2000). Moreover, for people in their working age, current society demands high adaptability, resistance to stress and continuous learning, which is impeded by cognitive dysfunction. This refrains many patients, even those with minor cognitive impairments, from holding employments at the level they were trained for. As amelioration of cognitive abilities could lead to improvements in daily life functioning, many studies have tried to improve cognitive functioning using various techniques (Bell & Bryson, 2001; Perneczky et al., 2006; Shin, Carter, Masterman, Fairbanks, & Cummings, 2005).

Current treatment options include rehabilitation, cognitive remediation, physical exercise, cognitive enhancing medication, and various brain stimulation techniques (Cicerone et al., 2005; Leroi, McDonald, Pantula, & Harbishettar, 2012; Wallace, Ballard, Pouzet, Riedel, & Wettstein, 2011), yet treatment effects vary and effect sizes (ES) are generally low. Non-invasive brain stimulation (NIBS) entails the modulation of brain excitability and activity (Ziemann et al., 2008) and consists of different methods, such as transcranial magnetic stimulation (TMS) and transcranial direct current stimulation (tDCS). TMS and tDCS have been used most commonly in an attempt to improve cognition in people with brain disorders. It is generally considered that anodal tDCS (AtDCS) increases the function of the underlying areas of the cortex, whereas cathodal tDCS has a suppressive effect (Nitsche & Paulus, 2011). TMS can either lead to an increase or decrease in cortical excitability depending on the stimulation frequency, varying from 1 to 50 Hertz. High tolerability with few side-effects is considered an important advantage over medication and, in the case of tDCS, has a potential to be applied at home (Aleman, Sommer, & Kahn, 2007, 2018; Chervyakov, Chernyavsky, Sinitsyn, & Piradov, 2015; Lage, Wiles, Shergill, & Tracy, 2016; Rossi et al., 2009; Slotema, Aleman, Daskalakis, & Sommer, 2012). Furthermore, contrasting cognitive remediation or practice and drill, NIBS demands little effort from patients, which is an important advantage for those who also suffer from fatigue, apathy, or diminished motivation.

Until now, the efficacy of NIBS is unclear and some have questioned whether these techniques have any effect on the brain at all (Vöröslakos et al., 2018). Skepticism is increased by lack of clear theories and evidence on working mechanisms of both interventions, which hampers smart applications (Chase, Boudewyn, Carter, & Phillips, 2020; Singh, Erwin-Grabner, Goya-Maldonado, & Antal, 2019) Nevertheless, many studies have applied NIBS techniques in an attempt to improve cognitive function in different diagnostic groups. Although some studies showed promising findings, the results of studies on the effect of NIBS on cognition remain inconsistent and the field would benefit from an overarching systematic overview of findings so far.

Cognition is a broad concept and different cognitive functions are subserved by different cerebral and cerebellar circuits, which may be more or less susceptible to stimulation techniques. As both TMS and tDCS are thought to affect mainly the outer layers of the brain, cognitive circuits that rely on midbrain and other deep structures such as the cingulate gyrus and the hippocampus may be expected to be insensitive to NIBS. In addition, since NIBS is usually applied to the cerebral cortex, cognitive functions that rely highly on subcortical or cerebellar circuits may not be expected to be responsive either. As the dorsolateral prefrontal cortex (DLPFC) is laterally located and often close to the area of stimulation, working memory may be a cognitive function expected to benefit from NIBS (Brunoni & Vanderhasselt, 2014; Miniussi & Rossini, 2011; Pope & Chris Miall, 2014; Tracy et al., 2015). Thus, the results of TMS and tDCS may become less heterogeneous when analyzed within the specific cognitive domains (attention/vigilance, working memory, executive functioning, processing speed, verbal fluency, verbal learning, and social cognition).

The aim of the current review and meta-analysis is therefore to quantitatively investigate the procognitive effect of NIBS (i.e. TMS and tDCS) in a domain-specific way, across different brain disorders in which cognitive dysfunction is a common problem. We evaluate all randomized controlled trials (RCTs) assessing the effect of the two most commonly applied types of NIBS (i.e. TMS and tDCS) for cognitive dysfunction in schizophrenia, depression, dementia, PD, MS, stroke, and TBI.

## Methods

### Search strategy

Following the Preferred Reporting for Systematic Reviews and Meta-analysis (PRISMA) Statement (Moher, Liberati, Tetzlaff, & Altman, 2009), a systematic search was performed in PubMed (Medline), EMBASE, Web of Science, and Cochrane Database of Systematic Reviews, using the following search terms: [‘cognition’ OR ‘cognitive functioning’] AND [‘transcranial magnetic stimulation’, ‘pulsed electromagnetic field therapy’, ‘low field magnetic stimulation’, ‘transcranial electrical stimulation’ OR ‘transcranial direct current stimulation’] AND [‘randomized controlled trial’, ‘RCT’ OR ‘randomized controlled study’], for each brain disorder (schizophrenia, depression, dementia, PD, MS, stroke, and TBI; exact terms described in online Supplementary Methods). No year or language limits were applied. Review articles and meta-analyses were examined for cross-references. The search cutoff date was 1 May 2019.

### Study inclusion

(1) RCTs investigating the effects of TMS or tDCS treatment on cognition measured with a neuropsychological test (battery);
(2) Studying patients affected by one of our conditions of interest;
(3) Single/double-blind studies comparing the treatment to a patient control group receiving sham stimulation. In case of combined interventions, the control group received the same non-brain stimulation component of the intervention (e.g. brain stimulation + medication *v.* sham + medication). Studies applying stimulation on a control stimulation site instead of sham were also included;
(4) Studies reporting sufficient information to compute common ES statistics [i.e. mean and standard deviations (s.d.), exact *F*-, *p*-, *t*, or *z*-values] or corresponding authors provided these data upon request;
(5) If multiple publications were retrieved describing the same cohort, the sample with the largest overall sample size and/or original data was included;
(6) Studies were published in an international peer-reviewed journal.

### Measures

For each identified study, we included pre- and post-assessments of cognitive functioning for the active *v.* sham condition. To facilitate cross-comparisons, cognitive outcomes were categorized into neurocognitive domains (Lage et al., 2016). We selected seven cognitive domains based on the two cognitive batteries commonly used in studies evaluating cognitive performance in psychiatric and neurological patient populations: the MATRICS (Measurement and Treatment Research to Improve Cognition in Schizophrenia; Green et al., 2004) and the Movement Disorder Society Task Force (Litvan et al., 2012): attention/vigilance, working memory, executive functioning, processing speed, verbal learning, verbal fluency, and social cognition. When a study applied multiple cognitive tests to assess the same cognitive domain, we included the primary outcome measure as defined by authors. When the primary outcome was not defined, we selected the test most relevant to the defined cognitive domain. If studies reported multiple outcomes for a single cognitive test (e.g. reaction time and number of errors), the outcome most relevant to the cognitive domain was included for analysis.

### Statistical analyses

We separately evaluated the effects of TMS and tDCS across the seven brain disorders, including all relevant study samples for the seven cognitive domains. Study samples were grouped according to brain disorder. We evaluated whether the effect varied per specific brain disorder and performed subanalyses when *k* ⩾ 3.

#### Calculations

All analyses were performed using Comprehensive Meta-Analysis Version 2.0 (Borenstein, Hedges, Higgins, & Rothstein, 2009). Details are provided in online Supplementary Methods. In short, Hedges' *g* was used to quantify ES for changes in cognitive performance, where a positive ES represented a superior effect of brain stimulation *v.* sham. Studies with multiple treatment groups (e.g. different stimulation intensity) and one sham group were entered as individual study samples (*k*). Single-dose (i.e. challenge) studies were included, sensitivity analyses were run with and without these challenge studies.

ES of *p* *<* 0.05 (two-tailed) were considered statistically significant, 0.2 reflecting a small, 0.5 a medium, and ⩾0.8 a large effect (Cohen, 1988). To investigate whether studies could be combined to share a common population ES, the *Q*-value and *I*^2^-statistic were evaluated. *I*^2^-values of 25, 50, and 75% are considered as low, moderate, and high heterogeneity, respectively (Cohen, 1988; Higgins, Thompson, Deeks, & Altman, 2003). Outlier studies were evaluated when heterogeneity was significant (*p* < 0.05), defined as standardized residual *Z*-scores of ES exceeding ±1.96 (*p* *<* 0.05, two-tailed).

For (trend-)significant results, potential publication bias was investigated by means of a visual inspection of the funnel plot and Egger's test (*p* *<* 0.1, two-tailed) (Egger, Smith, Schneider, & Minder, 1997). Rosenthal's fail-safe number (*N_R_*) was calculated, estimating the number of unpublished studies with non-significant results needed to bring an observed result to non-significance (*N_R_* ⩾ 5*k* + 10 to rule out a file drawer problem) (Rosenthal, 1979).

Sensitivity analyses were performed for (trend-)significant results, correcting for inflated control groups, single-dose studies, and moderator effects of the number of treatment sessions, mean age, and gender (online Supplementary Methods).

## Results

**Fig. 1.**
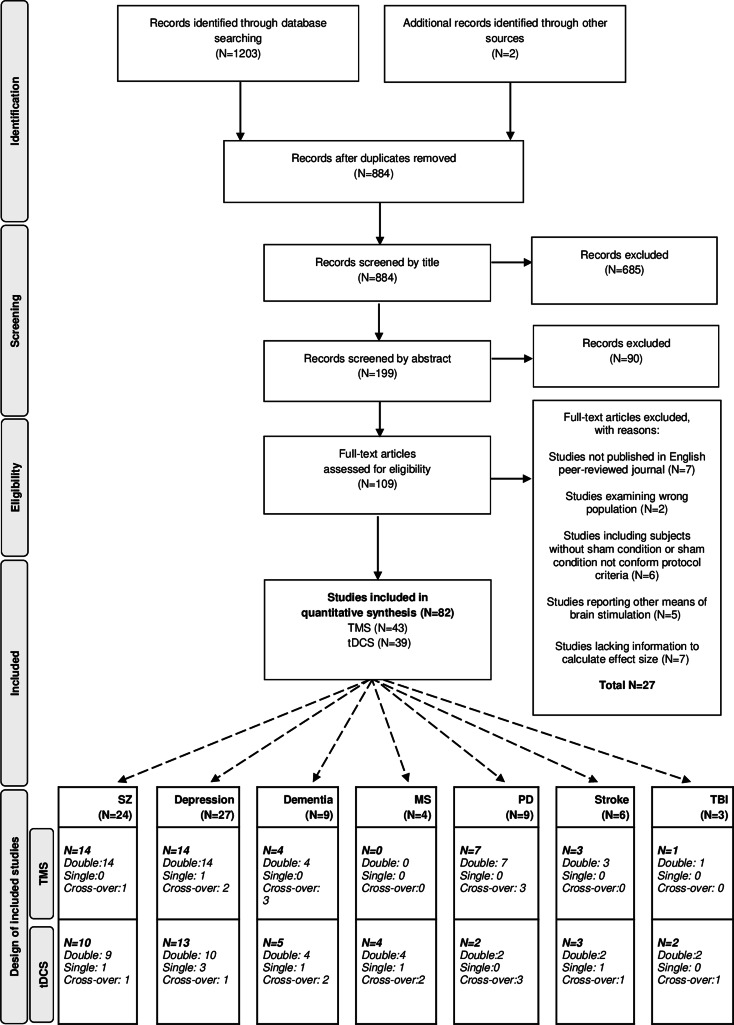
PRISMA flow diagram of the performed literature search. PRISMA, Preferred Reporting Items for Systematic Reviews and Meta-Analyses, dementia, depression, SZ (schizophrenia), MS (multiple sclerosis), PD (Parkinson's diseases), stroke and TBI (traumatic brain injury).

**Fig. 2.**
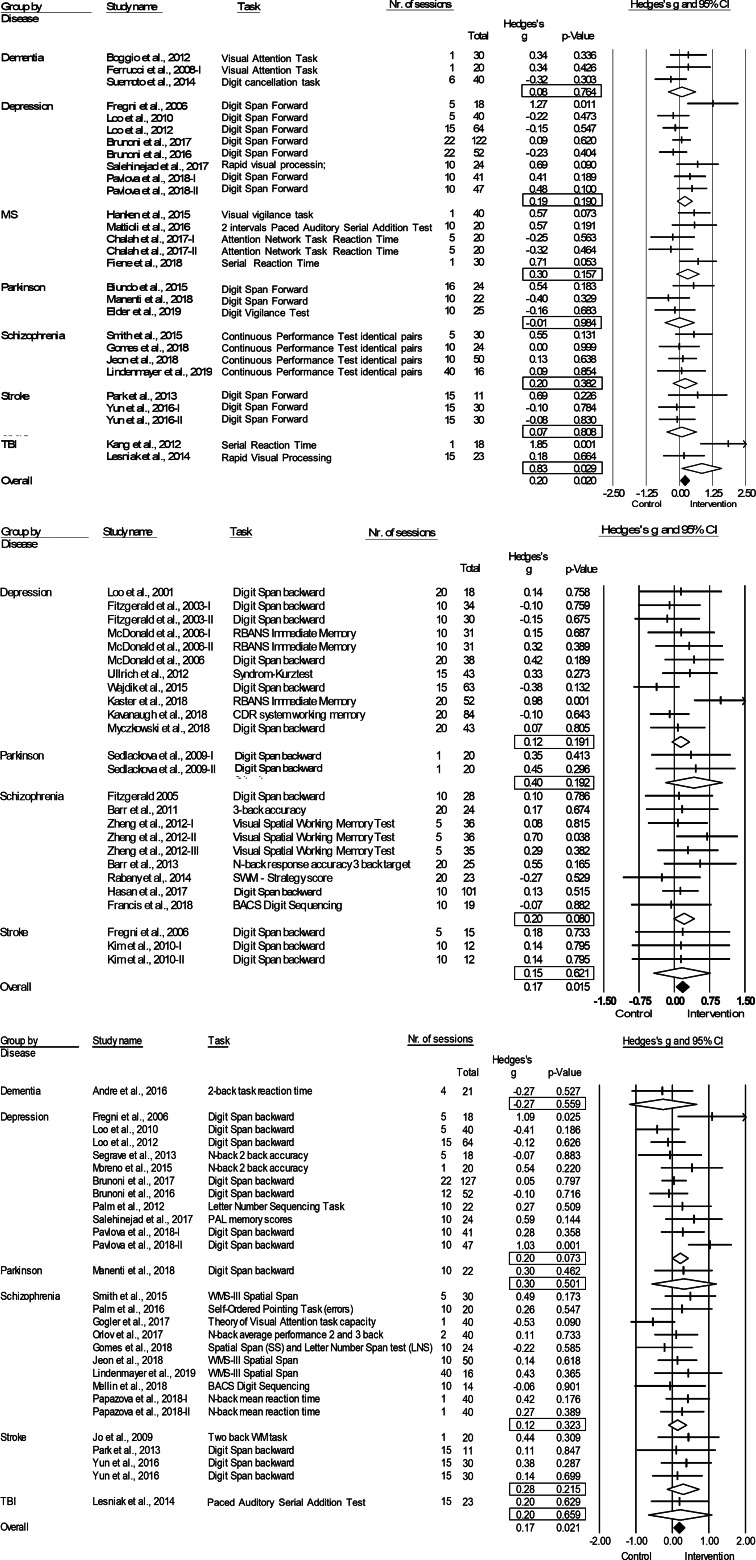
Forest plots of the effect of tDCS and TMS on working memory and tDCS on attention/vigilance. Results are summarized for all studies, sorted by brain disorder. (*a*) Forest plot of the effect of tDCS on attention/vigilance, outlier excluded. (*b*) Forest plot of the effect of TMS on working memory. (*c*) Forest plot of the effect of tDCS on working memory. BACS, Brief Assessment of Cognition in Schizophrenia; CDR, Cognitive Drug Research Computerized Assessment System; PAL, Paired Associate Learning; RBANS, Repeatable Battery for the Assessment of Neuropsychological Status; SWM, Spatial Working Memory; WMS, Wechsler Memory Scale; WM, Working Memory.

The literature search following PRISMA guidelines is depicted in Fig. 1. Demographic information on the included studies is provided in Table 1 for TMS and Table 2 for tDCS. The total number of included studies was 82, reporting on 93 study samples (*k*), evaluating a grand total of 2784 patients. Table 3 depicts the mean demographical group characteristics for the 43 TMS studies and 39 tDCS studies. Forest plots of the significant results are depicted in Fig. 2, forest plots of the remaining cognitive domains are depicted in online Supplementary Figs S1–S10.

**Table 1.** Characteristics of the included studies for Transcranial Magnetic Stimulation (TMS)

TMS study (year)		Design	Stimulation type/site	*n*	Age	%F	Duration of illness	Hz	Nr. of sessions	Cognitive domain(s)
*Schizophrenia*										
McIntosh et al. (2004)		Double	Left TPC	16	36 ± 10.9	56	AAO: 24 ± 6.0	1	4	VL
		Cross-over	Sham						4	
Fitzgerald et al. (2005)		Double	Left auditory TPC	17	16–65			1	10	A/V, WM, VF, VL
			Sham	16	16–65				10	
Mogg et al. (2007)		Double	Left DLPFC	8	51 ± 14.5	13	25y ± 16.7	10	10	EF, VF, VL
			Sham	9	34 ± 9.8	0	9y ± 7.9		10	
Mittrach et al. (2010)		Double	Left DLPFC	18	35 ± 0.5		6y ± 5.2	10	10	A/V, EF, PS
			Sham	14	34 ± 10.5	21	6y ± 8.8		10	
Barr et al. (2011)		Double	Bilateral DLPFC	12	47 ± 12.8	42		20	1	WM, PS
			Sham	12	47 ± 12.8	42			1	
Zheng, Guo, Li, Li, and Wang (2012)		Double	Left DLPFC	18	57 ± 5.4		AAO: 34 ± 6.5	20	5	WM, VF
			Left DLPFC	18	57 ± 7.4		AAO: 32 ± 7.2	10	5	
			ITBS; left DLPFC	19	56 ± 9.3		AAO: 33 ± 8.1	1–50	5	
			Sham	17	56 ± 5.8		AAO: 33 ± 10.0		5	
Barr et al. (2013)		Double	Bilateral DLPFC	13	41 ± 12.0	46	19y ± 11.7	20	20	WM, PS
			Sham	14	49 ± 12.4	21	25y ± 16.2		20	
Guse et al. (2013)		Double	Left pMFG	13	37 (22–58)	23	>0.5y	10	15	A/V, EF, PS
			Sham	12	36 (20–51)	25	>0.5y		15	
Wölwer et al. (2014)		Double	Left DLPFC	18	34 (22–59)	22	6y ± 5.2	10	10	A/V, EF, PS, SC
			Sham	14	34 (22–59)	21	6y ± 8.7		10	
Rabany, Deutsch, and Levkovitz (2014)		Double	Left DLPFC	20	33 ± 11.31	35	AAO: 21 ± 9.8	20	20	A/V, WM, EF, PS
			Sham	10	36 ± 11.0	20	AAO: 26 ± 8.3		20	
Dlabac-De Lange et al. (2015)		Double	Bilateral DLPFC	16	42 ± 11.6	13	16y ± 10.1	10	30	EF, PS, VF, VL
			Sham	16	32 ± 9.7	25	10y ± 8.9		30	
Wobrock et al. (2015)		Double	Left DLPFC	76	36 ± 10.5	18	>1y	10	15	EF, PS
			Sham	81	35 ± 9.1	31	>1y		15	
Hasan et al. (2016)		Double	Left DLPFC	77	36 ± 10.6	14	>1y	10	15	WM, EF, VF
			Sham	79	36 ± 9.0	28	>1y		15	
Francis et al. (2019)		Double	Bilateral DLPFC	9	23 ± 3.1	22	3y ± 1.6	20	10	VL, WM, EF, PS, VF
			Sham	10	22 ± 2.0	20	2y ± 1.1		10	
*Depression/bipolar*										
Loo et al. (2001)		Double	Left DLPFC	9	48	50		10	20	A/V, WM, EF, VL, VF
			Sham	9	48	50			20	
Moser et al. (2002)		Double	Left aMFG	9	61 ± 10.3			20	5	EF, PS, VF, VL
			Sham	10	61 ± 10.2				5	
Fitzgerald et al. (2003)		Double	HF; L PFC	20	42.20 ± 9.80	40	AAO: 29 ± 11.1	10	10	A/V, WM, EF, VL
			LF; R PFC	20	45.55 ± 11.49	35	AAO: 31 ± 14.9	1	10	
			Sham	20	49.15 ± 14.24	55	AAO: 34 ± 11.4		10	
Hausmann et al. (2004)		Double	HFL; DLPFC	12	47.33 ± 13.34	50		20	10	EF, PS, VF, VL
			HFL/LFR; DLPFC	13	45.23 ± 11.95	62		1–20	10	
			Sham	13	47.00 ± 11.31	69			10	
Mosimann et al. (2004)		Double	Left DLPFC	15	60.0 ± 13.4	33	AAO: 36 ± 16.7	20	10	EF, PS, VF, VL
			Sham	9	64.4 ± 13.0	56	AAO: 53 ± 14.0		10	
McDonald et al. (2006)		Double	HFL DLPFC	25	49 (41–55)	72		10	10	A/V, WM, VF
			LFR DLPFC	25	49 (39–54)	36		1	10	
			Sham	12	54 (47–64)	42			10	
Loo, Mitchell, McFarquhar, Malhi, and Sachdev (2007)		Double	Left PFC	19	49.8 ± 2.5	53	AAO: 28 ± 16.4	10	20	A/V, WM, PS, EF, VF, VL
			Sham	19	45.7 ± 15.0	42	AAO: 32 ± 13.2		20	
Vanderhasselt, de Raedt, Baeken, Leyman, and D'Haenen (2009)		Double	Left DLPFC	15	45.6 ± 5.87	60	AAO: 38 ± 16.4	10	10	EF
		Cross-over	Sham						10	
Ullrich, Kranaster, Sigges, Andrich, and Sartorius (2012)		Double	Left DLPFC	22	56.9 ± 10.2	69	7y ± 3.4	30	15	PS, WM
			Sham	21	54.1 ± 7.8	57	6y ± 6.0		15	
Wajdik et al. (2014)		Single	Left DLPFC	32	21–65			10	15	A/V, WM, EF, PS, VF, VL
			Sham	31	21–65				15	
Cheng et al. (2016)		Double	cTBS; right DLPFC	15	21–70			1–50	10	EF
			iTBS; left DLPFC	15	21–70			1–50	10	
			c + iTBS; bilateral DLPFC	15	21–70			1–50	10	
			Sham	15	21–70				10	
Kaster et al. (2018)		Double	DLPFC, VLPFC	25	65 ± 5.5	32	AAO: 33 ± 18.0	18	20	A/V, WM, EF, VF
			Sham	27	65 ± 5.5	44	AAO: 30 ± 18.6		20	
Kavanaugh et al. (2018)		Double	Left DLPFC	43	46 ± 11.6		NR	10	20	A/V, WM, PS
			Sham	41	48 ± 12.8				20	
Myczkowski et al. (2018)		Double	Left DLPFC	20	41 ± 11.7	75	12y ± 14.7	18	20	A/V, WM, EF, PS, VF, VL
			Sham	23	41 ± 9.0	78	11y ± 10.4		20	
*Dementia*										
Eliasova et al. (2014)		Double	IFG	10	75 ± 7.5	40	4 ± 1.6	10	2	EF, SP
		Cross-over	Sham						2	
Zhao et al. (2017)		Double	P3/P4, T5/T6	17	69 ± 5.8	59	NR	20	30	VL
			Sham	13	71 ± 5.2	54			30	
Koch et al. (2018)		Double	Precuneus	14	70 ± 5.1	50	14mo ± 5.1	20	10	EF, PS
		Cross-over	Sham						10	
Padala et al. (2018)		Double	Left DLPFC	4	68 ± 10.0	0	NR	10	20	EF, PS
		Cross-over	Sham	5	64 ± 9.0	20			10	
*Parkinson's disease*										
Sedláčková, Rektorová, Srovnalová, and Rektor (2009)	Double		Left PMCd	10	64 ± 6.7	10	8y ± 6.5	10	1	A/V, WM, PS
	Cross-over		Left DLPFC						1	
			Sham						1	
Pal, Nagy, Aschermann, Balazs, and Kovacs (2010)	Double		Left DLPFC	12	68.5 (median)	50	6y (median)	5	10	EF
			Sham	10	67.5 (median)	50	7y (median)		10	
Benninger et al. (2011)	Double		iTBS, Bilateral DLPFC	13	62 ± 6.9	46	11y ± 7.1	1–50	8	SP
			Sham	13	66 ± 9.0	15	7y ± 3.4		8	
Srovnalova, Marecek, and Rektorova (2011)	Double		Bilateral IFG	10	66 ± 6.0	40	5y ± 2.5	25	1	SP, EF
	Cross-over		Sham						1	
Benninger et al. (2012)	Double		Bilateral PMC	13	65 ± 9.1	15	9y ± 4.1	50	8	SP
			Sham	13	64 ± 8.3	31	9y ± 6.8		8	
Dagan, Herman, Mirelman, Giladi, and Hausdorff (2017)	Double		Mpfc	7	76 ± 6.47	0	10y ± 3.8	10	8	EF
	Cross-over		Sham	6			10y ± 3.8		8	
Buard et al. (2018)	Double		Bilateral DLPFC	22	67 ± 7.2	27		20	10	A/V, EF, PS, VL, VF
			Sham	24	70 ± 8.0	29			10	
*Stroke*										
Fregni, Boggio, Nitsche, Rigonatti, and Pascual-Leone (2006a, 2006b)	Double		PMC unaffected side	10	58 ± 11.3	20	4y ± 2.9	1	5	A/V, WM, EF, PS
			Sham	5	53 ± 12.6	40	4y ± 2.6		5	
Kim, Kim, Ho Chun, Hwa Yi, and Sung Kwon (2010)	Double		HF; left DLPFC	6	54 ± 16.9	33	241da ± 42.5	10	10	A/V, WM, EF, PS, VL
			LF; left DLPFC	6	68 ± 7.4	67	404da ± 71.7	1	10	
			Sham	6	67 ± 17.2	33	70da ± 39.0		10	
Sun (2015)	Double		Right DLPFC	22	43 ± 12.3	37	67da (30–365)	1	20	VL
			Sham	22	47 ± 11.8	38	56da (30–296)		20	
*Traumatic Brain Injury*										
Lee and Kim (2018)	Single		Right DLPFC	7	42 ± 11.3	29	4mo ± 1.7	1	10	EF
			Sham	6	41 ± 11.0	33	4mo ± 1.9		10	
										

Hz, Hertz; DLPFC, dorsolateral prefrontal cortex; IFG, inferior frontal gyrus; MFG, middle frontal gyrus; PMC, premotor cortex; TPC, temporo-parietal cortex; VLPFC; ventrolateral prefrontal cortex; a, anterior; m, medial; p, posterior; d, dorsal; HF, high frequency; LF, low frequency; iTBS, intermitted theta burst stimulation; cTBS, continuous theta burst stimulation; c + iTBS, continuous and intermittent theta burst stimulation; AAO, age at onset; y, years; mo, months; da, days; A/V, attention/vigilance; EF, executive functioning; PS, processing speed; VF, verbal fluency; VL, verbal learning; WM, working memory.

Year of publication (Year), study design (Design), stimulation type and/or site, number of participants per group (*n*), age (mean ± standard deviation), proportion of females (%F), duration of illness (mean ± standard deviation if available), stimulation frequency (Hz), number (nr.) of sessions and cognitive domains(s) are specified for each study.

**Table 2.** Characteristics of the included studies for transcranial direct current stimulation (tDCS)

tDCS study (year)	Design	Stimulation type/site	*n*	age ± s.d.	%F	Duration of illness	mA	Nr. of sessions	Cognitive domain(s)
*Schizophrenia*									
Smith et al. (2015)	Double	A: left DLPFC, C: right SO ridge	17	47 ± 11.1	18		2	5	A/V, WM, EF, PS, VL, SC
		Sham	16	45 ± 9.2	38			5	
Rassovsky et al. (2015)	Single	A: DLPFC, C: right SO	12	46 ± 11.2	17	20y ± 13.8	2	1	SC
		C: DLPFC, A: right SO	12	48 ± 7.5	50	25y ± 10.9		1	SC
		Sham	12	42 ± 10.3	33	15y ± 7.7		1	
Palm et al. (2016)	Double	A: left DLPFC, C: right SO	10	38 ± 12.9	50	7y ± 6.1	2	10	WM, EF, PS,
		Sham	10	34 ± 10.7	0	14y ± 12.1		10	
Gögler et al. (2017)	Double	A: left DLPFC, C: right SO	20	37 ± 9.2	35	7y ± 5.9	2	1	WM, EF, PS
		Sham	20	32 ± 8.3	50			1	
Orlov et al. (2017)	Double	A: left DLPFC, C: right SO	22	35 ± 9.4	14	13y ± 7.3	2	2	WM, VL
		Sham	25	39 ± 9.4	16	17y ± 9.2		2	
Gomes et al. (2018)	Double	A: left DLPFC, C: right DLPFC	12	39 ± 9.3	17	16y ± 11.6	2	10	A/V, WM, EF, PS, VL
		Sham	12	34 ± 12.1	42	10y ± 7.3		10	
Jeon et al. (2018)	Double	A: left DLPFC, C: right DLPFC	25	40 ± 9.4	50	13y	2	10	A/V, WM, EF, PS, VL, SC
		Sham	27	40 ± 12.4	57	14y		10	
Mellin et al. (2018)	Double	A: Left DLPFC, C: left TPJ	7	30 ± 11.0			2	10	WM, EF, PS, VF, VL
		Sham	7	39 ± 10.0				10	
Papazova et al. (2018)	Double	A: left DLPFC, C: right deltoid muscle	20	37 ± 10.6	45	9y ± 7.6y	1	1	WM
	Cross-over	A: left DLPFC, C: right deltoid muscle					2	1	
		Sham						1	
Lindenmayer et al. (2019)	Double	A: left DLPFC, C: left AC	15	40 ± 10.7	13	3y ± 4.5	2	40	A/V, WM, EF, PS, VL, SC
		Sham	13	40 ± 10.7	15	3y ± 4.5		40	
*Depression/bipolar*									
Fregni et al. (2006a, 2006b)	Double	A: left DLPFC, C: right SO	9	48 ± 10.4	56	10y ± 5.9	1	5	A/V, WM, EF, PS
		Sham	9	45 ± 9.3	67	9y ± 4.2		5	
Boggio et al. (2007)	Double	A: left DLPFC, C: right SO	12	48 ± 9.9	50	24y ± 7.4	2	1	SC
		Sham	7	47 ± 10.4	71	22y ± 10.6		1	
Loo et al. (2010)	Double	A: left DLPFC C:right lat. O	20	49 ± 10.0	55	AAO: 31y ± 14.1	1	5	A/V, WM, EF, PS, VF, VL
		Sham	20	46 ± 12.5	55	AAO: 32y ± 14.7		5	
Loo et al. (2012)	Double	A: left DLPFC, C:right lat. O	31	48 ± 12.5	45	AAO: 28y ± 12.6	2	15	A/V, WM, EF, VF, VL
		Sham	29	49 ± 12.6	48	AAO: 28y ± 12.5		15	
Palm et al. (2012)	Double	A: left DLPFC, C: right SO; active first	11	56 ± 12.0	55	AAO: 44y ± 10.0	0.5	10	WM, VF, VL
	Cross-over^a^	A: left DLPFC, C: right SO; sham first	11	58 ± 12.0	73	AAO: 4y ± 15.0	0.5	10	
Segrave, Arnold, Hoy and Fitzgerald (2014)	Double	A: left DLPFC, C: right lat. O; CCT	9	43 ± 18.3	22	AAO: 17y ± 9.1	2	5	WM, PS
		Sham; CCT	9	45 ± 10.2	44	AAO: 16y ± 5.9		5	
Bennabi et al. (2015)	Double	A: left DLPFC, C: right SO	12	60 ± 12.0	83		2	10	PS, EF, VL
		Sham	11	60 ± 5.4	46			10	
Moreno et al. (2015)	Single	A: left DLPFC, C: right DLPFC	10	35 ± 4.1	50		2	1	WM, PS, SC
		Sham	10	32 ± 4.7	50			1	
Brunoni et al. (2016)	Double	A: left DLPFC, C: right DLPFC	26	41 ± 12.0	81		2	12	A/V, WM, EF, PS
		Sham	26	46 ± 14.0	77			12	
Bersani et al. (2017)	Double	A: left DLPFC, C: right CBC	21	48 ± 10.7	71	19y ± 11.0	2	15	EF, PS
		Sham	21	29 ± 10.2	38	12y ± 6.6		15	
Brunoni et al. (2017)	Double	A: left DLPFC, C: right DLPFC; oral placebo	72	45 ± 11.8	68	AAO: 26y ± 11.7	2	22	A/V, WM, EF, PS, VF
		Sham; oral placebo	55	41 ± 12.9	68	AAO: 26y ± 11.3		22	
Salehinejad, Ghanavai, Rostami, and Nejati (2017)	Single	A: left DLPFC, C: right DLPFC	12	27 ± 7.1	58		2	10	A/V, WM
		Sham	12	26 ± 4.6	67			10	
Pavlova et al. (2018)	Single	A: left DLPFC, C: right SO; 20 min	21	36 ± 10.8	81		0.5	10	A/V, WM, PS, VF
		A: left DLPFC, C: right SO; 30 min	27	37 ± 8.8	63		0.5	10	
		Sham	20	40 ± 12.2	75			10	
*Dementia*									
Ferrucci et al. (2008)	Double	A: TP area, C: right deltoid muscle	10	75 ± 7.3	70		1.5	1	A/V
	Cross-over	Sham			70			1	
Boggio et al. (2012)	Double	A: bilateral TC; C: right deltoid muscle	15	79 ± 8.1	40	4y ± 1.7	2	5	A/V, VL
	Cross-over	Sham				4y ± 1.7		5	
Suemoto et al. (2014)	Double	A: left DLPFC, C: right SO	20	79 ± 7.1	75		2	6	A/V, WM
		Sham	20	82 ± 8.0	65			6	
Biundo et al. (2015)	Double	A: left DLPFC, C: right SO	7	69 ± 7.6	14		2	10	A/V, PS, VF, VL
		Sham	9	72 ± 4.1	11			10	
André et al. (2016)	Single	A: left DLPFC, C: right SO	13	63–94			2	1	EF, WM
		Sham	8	63–94				1	
*Parkinson's disease*									
Manenti et al. (2018)	Double	A: left DFPFC, C: right SO; CT	11	66 ± 6.4	55	6y ± 3.9	2	10	A/V, EF, PS, VF, VL
		Sham; CT	11	64 ± 7.1	36	8y ± 3.4		10	
Elder, Colloby, Firbank, McKeith, and Taylor (2019)	Double	A: right PC; C: OcC	19	76 ± 8.8	21		1.2	10	PS
		Sham	17	74 ± 7.0	29			10	
*Stroke*									
Jo et al. (2009)	Single	A: left DLPFC, C: right SO	10	48 ± 8.7	30	72da ± 30.0	2	1	WM
	Cross-over	Sham						1	
Yun, Chun, and Kim (2015)	Double	A: left aTL; C: right SO	15	61 ± 12.9	60	42da ± 31.9	2	15	A/V, WM, PS, VL
		A: right aTL, C: left SO	15	59 ± 15.0	53	38da ± 27.0	2	15	
		Sham	15	69 ± 14.6	53	40da ± 29.6		15	
Park, Koh, Choi, and Ko (2013)	Double	A: bilateral PFC, C: non-dominant arm	6	65 ± 14.3	67	29da ± 18.7	2	15	A/V, WM, PS
		Sham	5	66 ± 10.8	40	25da ± 17.5		15	
*Traumatic brain injury*									
Kang, Kim, and Paik (2012)	Double	A: left DLPFC, C: right SO	9	50 ± 7.2	11	18mo ± 4.3	2	1	A/V
	Cross-over	Sham						1	
Leśniak, Polanowska, Seniów, and Członkowska (2014)	Double	A: left DLPFC, C: right SO; CT	14	28 ± 9	33	11mo ± 5.8	1	15	A/V, WM, VL
		Sham; CT	12	29 ± 7.7	18	13mo ± 6.4		15	
*Multiple sclerosis*									
Hanken et al. (2016)	Double	A: right PC, C: left forehead	20	51 ± 9.4	65		1.5	1	A/V
		Sham	20	49 ± 9.7	40			1	
Mattioli et al. (2016)	Double	A: left DLPFC, C: right shoulder	10	38 ± 10.0	70	7y ± 6.1	2	10	A/V, EF, PS, VL
		Sham	10	47 ± 10.4	90	11y ± 6.5		10	
Chalah et al. (2017)	Double	A: left DLPFC, C: right SO	10	41 ± 11.2	40	14y ± 9.9	2	5	EF
	Cross-over	A: right pPC, C: Cz						5	
		Sham						5	
Fiene et al. (2018)	Single	A: left DLPFC C: right shoulder	15	43 ± 15.0	53	10y ± 8.6	1.5	1	A/V
	Cross-over	Sham						1	

mA, miliÀmpère; A, anodal; C, cathodal; CT, cognitive therapy; CCT, cognitive control therapy; AC, auditory cortex; CBC; cerebellar cortex; Cz, central midline; DLPFC, dorsolateral prefrontal cortex; IFG, inferior frontal gyrus; lat. O, lateral aspect of the orbit; OcC, occipital cortex; PC, parietal cortex; PMC, premotor cortex; SO, supraorbital area; TC, temporal cortex; TL, temporal lobe; TP, temporo-parietal; TPJ, temporo-parietal junction; VLPFC, ventrolateral prefrontal cortex; a, anterior; m, medial; p, posterior; min, minutes; AAO, age at onset; y, years; mo, months; da, days; A/V, attention/vigilance; EF, executive functioning; PS, processing speed; SC, social cognition; VF, verbal fluency; VL, verbal learning; WM, working memory.

Year of publication (Year), study design (Design), stimulation type and/or site, number of participants per group (*n*), age (mean ± standard deviation), proportion of females (%F), duration of illness (mean ± standard deviation if available), stimulation intensity (mA), number (nr.) of sessions and cognitive domains(s) are specified for each study.

a Within-group cross-over.

**Table 3.** Mean group characteristics for the included TMS and tDCS studies

	TMS	tDCS
Number of studies included, *N*	43	39
Number of study-samples included, *k*	49	44
Participants, total *n*	1591	1193
Study-sample size, mean (s.d.)	18 (±15.0)	19 (±15.2)
Age in years, mean (s.d.)	49.0 (±9.88)	45.7 ± 10.91
Gender, proportion of females (s.d.)	37 (±18.2)	48 (±21.5)

### Attention/vigilance

*TMS:* Combining 21 study samples (*n* = 680), TMS did not differ from sham in the effect on attention/vigilance, studies were homogeneous (ES = 0.10, *p* = 0.210; Table 4). ES did not differ between the four different brain disorders included (online Supplementary Table S1).

**Table 4.** Effects of Transcranial Magnetic Stimulation (TMS) and transcranial Direct Current Stimulation (tDCS) across brain disorders for the seven cognitive domains

Cognitive domain	Type	*k*	*n*	Hedges' *g*	95% CI	*p* value	*Q*-statistic (df)	*I*^2^ (%)	*N_R_*
Attention/Vigilance	TMS	21	679	0.10	(−0.078 to 0.263)	0.210	*Q*(20) = 19.15, *p* = 0.512	0	
	tDCS	29	980	**0.27**	**(0.074 to 0.457)**	**0.006**	***Q*(28) = 58.43, *p* = 0.001**	52.08	93
	tDCS without outlier	28	931	**0.20**	**(0.032 to 0.367)**	**0.020**	*Q*(27) = 39.64; *p* = 0.055	31.90	45
Working Memory	TMS	25	873	**0.17**	**(0.030 to 0.299)**	**0.015**	*Q*(24) = 22.47, *p* = 0.551	4.10	18
	tDCS	28	939	**0.17**	**(0.026 to 0.319)**	**0.021**	*Q*(27) = 29.90, *p* = 0.319	9.70	28
Executive Functioning	TMS	40	1145	0.07	(−0.046 to 0.184)	0.243	*Q*(39) = 38.36, *p* *=* 0.499	0	
	tDCS	19	662	0.04	(−0.201 to 0.288)	0.726	***Q*(18) = 36.52, *p* *=* 0.006**	50.72	
	tDCS without outliers	17	622	0.00	(−0.154 to 0.156)	0.992	*Q*(16) = 11.51, *p* *=* 0.777	0	
Processing Speed	TMS	30	941	−0.01	(−0.134 to 0.118)	0.900	*Q*(29) = 23.71, *p* *=* 0.743	0	
	tDCS	24	754	0.24	(−0.030 to 0.565)	0.099	***Q*(23) = 76.87, *p* < 0.05**	70.55	
	tDCS without outliers	21	732	−0.02	(−0.163 to 0.123)	0.784	*Q*(20) = 20.71, *p* = 0.414	3.45	
Verbal Fluency	TMS	21	747	−0.05	(−0.187 to 0.097)	0.538	*Q*(20) = 14.12, *p* = 0.825	0	
	tDCS	9	399	0.14	(−0.170 to 0.351)	0.193	*Q*(8) = 7.26, *p* = 0.509	0	
Verbal Learning	TMS	21	690	0.08	(−0.147 to 0.241)	0.635	***Q*(20) = 32.21, *p* = 0.041**	37.91	
	TMS without outliers	20	651	−0.01	(−0.165 to 0.139)	0.866	*Q*(19) = 17.82, *p* = 0.534	0	
	tDCS	18	551	0.05	(−0.137 to 0.233)	0.609	*Q*(17) = 16.44, *p* = 0.493	0	
Social Cognition	tDCS	7	171	0.27	(−0.023 to 0.566)	0.070	*Q*(6) = 1.72, *p* = 0.190	0	2

*k*, number of study samples; *n*, number of studies; df, degrees of freedom; *I^2^*, heterogeneity; *N_R_*, Rosenthal's Fail-safe number.

*Note*: Bold values indicate significant test-statistic (*p* < 0.05).

*tDCS:* tDCS (*k* = 29; *n* = 980) was superior to sham in improving performance on attention/vigilance tasks (ES = 0.27, *p* *=* 0.006; Table 4; Fig. 2*a*). The number of null-studies needed to render this effect non-significant (*N_R_*) was 93, with no evidence for publication bias (online Supplementary Fig. S11). However, heterogeneity was moderate and the *I^2^* of 52.08 indicated that 48.92% of the dispersion reflects the difference in the true ES while the remaining 52.08% can be attributed to random sampling error (Table 4). One outlier study was identified (*Z*-score>1.96; Orlov et al., 2017). This pilot study combined two sessions on day 1 and day 14, with four sessions of cognitive therapy in-between. After exclusion, ES remained significant and heterogeneity decreased (ES = 0.20, *p* *=* 0.020; Table 4; Fig. 2*b*). However, the funnel plot indicated potential publication bias (*p* = 0.047, online Supplementary Fig. S11). The effect of tDCS did not differ across disorders (online Supplementary Table S1). Regarding the sensitivity analyses, four studies compared multiple interventions to the same control group, yet the mean weighted ES did not change significantly after splitting these shared placebo groups to prevent inflated ES (ES = 0.20, *p* *=* 0.022). However, when excluding challenge/single-dose studies, the effect of tDCS on attention/vigilance was no longer significant (ES = 0.12, *p* *=* 0.140). Meta-regressions did not reveal significant moderation effects for the number of treatment sessions, age, or gender (online Supplementary Table S2).

### Working memory

**Fig. 3.**
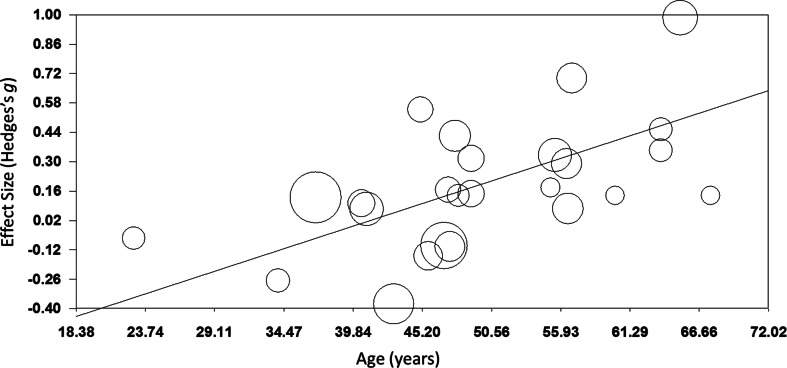
Meta-regression of the effect of TMS on working memory. Study-samples depicted by circles proportional to their sample size. The *x*-axis represents the mean age of the study-samples in years, *y*-axis depicts the effect size (Hedges' *g*).

*TMS:* TMS (*k* = 25; *n* = 873) showed a small yet significant effect, with low heterogeneity (ES = 0.17, *p* *=* 0.015; Table 4; Fig. 2*b*). ES did not differ between brain disorders (online Supplementary Table S1) and publication bias was low (online Supplementary Fig. S12). This effect of TMS on working memory did not change after correcting control group sample sizes of the five studies that compared multiple interventions (ES = 0.16, *p* *=* 0.032) or after excluding one single-dose study (ES = 0.15, *p* *=* 0.049). Notably, age was a positive moderator, as study samples with a higher mean age showed more improvement in working memory after TMS in a linear fashion as depicted in Fig. 3 (slope coefficient *=* 0.020, *p* = 0.005). The number of treatment sessions and gender did not moderate ES (online Supplementary Table S2).

*tDCS:* A small significant effect on working memory was detected for tDCS, studies were homogeneous (*k* = 28; *n* = 939; ES = 0.17, *p* *=* 0.021; Table 4; Fig. 2*c*) and there was no evidence for publication bias (online Supplementary Fig. S13). This effect did not differ across different brain disorders (online Supplementary Table S1). ES remained significant when adjusting control group sample sizes for the three studies with multiple intervention groups (ES = 0.15, *p* *=* 0.038) and when excluding five single-dose studies (ES = 0.17, *p* *=* 0.038). No significant moderators were identified (online Supplementary Table S2).

### Executive functioning

*TMS:* Overall, TMS (*k* = 40; *n* = 1145) was not superior to sham in improving executive functioning, with low heterogeneity (ES = 0.07, *p* = 0.243; Table 4), ES did not differ between disorders (online Supplementary Table S1).

*tDCS:* tDCS (*k* = 19, *n* = 662) did not differ from sham, with moderate heterogeneity (ES = 0.04, *p* = 0.726; Table 4). ES did not differ across disorders (online Supplementary Table S1). Mattioli, Bellomi, Stampatori, Capra, and Miniussi (2016) and the DLPFC-condition of Chalah, Créange, Lefaucheur, and Ayache (2017) were both identified as outliers. Mattioli et al. (2016) was a positive outlier (*Z*-score >1.96) and the only study using total errors of the Wisconsin's Card Sorting Test as an outcome measure. In line with the positive effect that was found for right PPC stimulation in the same study (Table 1), Chalah et al. (2017) relate their strong negative effect (*Z*-score <1.96) to the phenomenon of cerebral lateralization in MS. After exclusion, heterogeneity decreased and ES remained non-significant (ES = 0.00, *p* = 0.992; Table 4).

### Processing speed

*TMS:* No superior effect was found for TMS (*k* = 30; *n* = 941) *v.* sham, with low heterogeneity (ES *=* −0.01, *p* *=* 0.900; Table 4), without any differences across the included disorders (online Supplementary Table S1).

*tDCS:* No difference was found in the effect of tDCS (*k* = 24; *n* = 794) *v.* sham (ES = 0.24, *p* *=* 0.099, Table 4), ES did not vary between disorders (online Supplementary Table S1). Heterogeneity was moderate to high (Table 4), three outlier studies were identified. Segrave et al. (2014) reported remarkable strong improvements after sham compared to tDCS (*N*-back RT, *Z*-score <−1.96). Diverging from other included studies, both Moreno et al. (2015) (*Z*-score >1.96) and Biundo et al. (2015) (*Z*-score >1.96) used the RBANS written coding task. After exclusion, heterogeneity decreased and ES remained non-significant (ES = −0.02, *p* = 0.784; Table 4). Notably, the effect of tDCS then varied between different brain disorders [*Q*(4) = 12.62; *p* = 0.013; online Supplementary Table S1] yet subgroup analyses showed that tDCS was not superior for depression (*k* = 8; *n* = 389; ES = −0.00, 95% CI −0.20 to 0.20, *p* = 0.997), schizophrenia (*k* = 7; *n* = 194; ES = −0.02, 95% CI −0.29 to 0.26, *p* = 0.894), and stroke (*k* = 3; *n* = 71; ES = 0.10, 95% CI −0.35 to 0.55, *p* = 0.654). The number of studies on PD (*k* = 2) and MS (*k* = 1) was insufficient to perform subgroup analyses.

### Verbal fluency

*TMS:* No superior effect was found for TMS (*k* = 21; *n* = 747) on verbal fluency, with low heterogeneity (ES = −0.05, *p* = 0.538; Table 4) and no differences across disorders (online Supplementary Table S1).

*tDCS:* No significant difference was found between tDCS (*k* = 9; *n* = 399) *v.* sham, heterogeneity was low (ES = 0.14, *p* = 0.193; Table 4), ES did not vary across disorders (online Supplementary Table S1).

### Verbal learning

*TMS:* Overall, TMS (*k* = 21; *n* = 690) showed no positive effect *v.* sham but heterogeneity was high (ES = 0.08, *p* = 0.635; Table 4). Significant differences in effect between disorders were detected (*p* = 0.029; online Supplementary Table S1), subgroup analyses showed an effect for stroke [*k* = 3; *n* = 64; ES = 0.677, Q(2) = 7.01, *p* = 0.005]. After excluding one outlier study (*Z*-score >1.96) (Sun, Lu, Zhang, Wen, & Sun, 2015), heterogeneity decreased and overall ES remained non-significant (Table 4). Notably, the difference in the effect of TMS on verbal learning between disorders was then no longer observed (online Supplementary Table S1).

*tDCS:* tDCS did not differ from sham (*k* = 18; *n* = 550), included studies were homogeneous (ES = 0.05, *p* = 0.609; Table 4) and ES did not differ across disorders (online Supplementary Table S1).

### Social cognition

*TMS:* As we retrieved only one study investigating the effect of TMS on social cognition (Wölwer et al., 2014), no meta-analysis could be conducted.

*tDCS:* tDCS (*k* = 7; *n* = 171) showed a trend-significant effect on social cognition, with low heterogeneity (ES = 0.27, *p* = 0.070; Table 4). The funnel plot and Egger's regression test indicated potential publication bias (*p* = 0.09; online Supplementary Fig. S13), yet the power of this test is limited due to the small number of studies included (Egger et al., 1997). The difference in ES between disorders was trend-significant (online Supplementary Table S1). The studies on schizophrenia showed a non-significant effect (*k* = 5; *n* = 132; ES = 0.17, *p* = 0.326), although the two studies on depression were positive they could not be combined in meta-analysis.

## Discussion

We investigated the procognitive effects of TMS and tDCS in 83 randomized, double- or single-blind sham-controlled studies in schizophrenia, depression, dementia, MS, PD, stroke, and TBI. Using a domain-specific approach, we found a positive effect of both TMS (ES = 0.17) and tDCS (ES = 0.17) as compared to sham on working memory. The finding that both NIBS techniques elicit a similar effect in the same domain may suggest that the circuitry underlying working memory is readily accessible for activation using NIBS. In addition, this is the first meta-analysis reporting improvements in attention/vigilance in tDCS as compared to placebo stimulation (ES = 0.19), while the ES of 0.10 for TMS was not significant. This suggests that tDCS may be superior to TMS in improving attention/vigilance, although firm conclusions can only be drawn by making direct comparisons within one study randomizing participants across conditions. Other cognitive domains did not benefit from either TMS or tDCS as compared to sham.

Our findings largely corroborate with two quantitative reviews that have specifically evaluated the effects of NIBS on working memory, both including a lower number of studies (Brunoni & Vanderhasselt, 2014; Hill, Fitzgerald, & Hoy, 2016; Martin, McClintock, Forster, & Loo, 2016). Hill et al. (2016) (16 AtDCS studies; 182 neuropsychiatric patients and 170 controls) showed that AtDCS significantly improved online accuracy in patients (ES = 0.77) and offline reaction time in healthy individuals (ES = 0.16) as compared to sham, although no overall effect was found for accuracy or reaction time. Brunoni and Vanderhasselt (2014) (12 TMS and tDCS RCTs, 805 psychiatric patients and healthy controls) identified medium effects of NIBS, on accuracy (ES correct responses = 0.25; ES error rates = 0.29) and reaction time (ES = −0.22), yet meta-regressions revealed that accuracy only improved in participants receiving TMS and not tDCS. Both of the abovementioned reviews did not evaluate differences across disorders. Moreover, Hill et al. (2016) only selected studies that applied the *N*-back, Sternberg, or digit-span task, while Brunoni and Vanderhasselt (2014) included NIBS studies specifically targeting the DLPFC and implementing the *N*-back task. A third review by Martin et al. (2016) evaluating multiple cognitive domains reported a significant ES of 0.51 (95% CI 0.18–0.83) after pooling three rTMS studies for working memory in secondary analyses.

Interestingly, we found a significant moderator effect of age for TMS on working memory, indicating that the potential procognitive effect of TMS is larger at a higher age. A possible explanation for this finding could be that the skull thickness decreases over age, making it easier to stimulate the cortex underneath due to a shorter distance of the cortex from the coil. However, this finding is not supported by conventional theories emphasizing that the distance between the cortex and the coil increases with age due to brain atrophy, negatively affecting TMS efficacy (McConnell et al., 2001). Also, there might be more potential for cognitive improvement in an older population as younger patients may show ceiling effects in cognitive functioning (Lillie, Urban, Lynch, Weaver, & Stitzel, 2016; Schulte-Geers et al., 2011). Although we found no differences in effect between disorders, the effect of age might be an indirect reflection of subtle differences in treatment effect between disorders. Where dementia and PD usually strike at a high age, schizophrenia and MS overall affect a relatively young population. Furthermore, the diseases with late age of onset generally have more detrimental effects on cognition. More research is needed to gain more insight into the potential associations between TMS efficacy and age and potential ceiling effects to explain this unexpected effect.

One of the possible mechanisms behind the positive effects of NIBS on working memory and of tDCS on attention/vigilance may include an increase in dopamine release. Recently, Fonteneau et al. (2018) have demonstrated that a single session of bifrontal tDCS induced dopamine release in the ventral striatum in healthy individuals (*n* = 32). Striatal dopamine function not only links to neural efficiency of the (dorsolateral) striatum but also of the prefrontal cortex and associated higher-order cognitive functioning, including attention switching and working memory updating (Cools, 2011; Landau, Lal, O'Neil, Baker, & Jagust, 2009; Stelzel, Basten, Montag, Reuter, & Fiebach, 2010). For the five other cognitive domains investigated in the present meta-analysis, we did not find any procognitive effects of either TMS or tDCS. It is possible that these domains rely more on subcortical, cerebellar, medial, and fusiform areas, being not directly accessible using NIBS techniques (Demirtas-Tatlidede, Vahabzadeh-Hagh, & Pascual-Leone, 2013; Kim, Hong, Kim, & Yoon, 2019). It is also possible that a longer duration of stimulation or a higher stimulation frequency or intensity is required to influence these domains. Alternatively, a combination of cognitive training and NIBS may be needed to gain improvement. Although not included in the present meta-analysis, a positive effect of NIBS on global cognitive function and verbal fluency was recently demonstrated in a quantitative review on patients with mild cognitive impairment (Xu et al., 2019) in addition to a small positive effect on executive function (11 RCTs, 367 patients).

Overall, we found no evidence for the effect of either TMS or tDCS to differ across brain disorders. Although TMS showed a positive effect on verbal learning for stroke only (*k* = 3) and tDCS on processing speed for MS (*k* = 1) and PD (*k* = 2) only, no definite conclusions could be drawn regarding the nature of these differences due to the limited number of studies that could be included for the relevant brain disorders. The existence of common shared pathophysiologic substrates regarding decreased plasticity across brain disorders has been proposed to underlie cognitive decline in different brain disorders, which may suggest that patients with different brain disorders could benefit from the same procognitive interventions (Demirtas-Tatlidede et al., 2013; Kim et al., 2019), which has indeed been shown to be the case for exercise (Dauwan et al., 2019; Herold, Törpel, Schega, & Müller, 2019).

### Strengths and limitations

An important strength of this study is the inclusion of seven different cognitive domains that were analyzed separately, uncovering the differences in effects across different domains. In our opinion, this approach is relevant, as cognition is subserved by different cerebral and cerebellar circuits. Different circuits in the brain are responsible for the proper functioning of different domains, which follows that the effect of NIBS may be inconsistent across domains. This inconsistency has been reported in prior studies (Lindenmayer et al., 2019; Loo et al., 2010; Manenti et al., 2018). Splitting up cognition into relevant separate domains elucidates effects that might be hidden when looking at global cognition.

Also, this meta-analysis provides an answer to the need for a comparison between the two most commonly used types of NIBS. The relevance of such a comparison is supported by the existing debate about which of the two is the most effective and suitable for clinical use (Brunoni & Vanderhasselt, 2014; Inukai et al., 2016). Our meta-analysis reveals that both seem to have a profound effect on a specific and corresponding cognitive domain, namely working memory. Although tDCS also impacted attention/vigilance, the effects of both types are nonetheless absent in the other domains. While tDCS and TMS are different types of stimulation with different working mechanisms, our findings indicate that they might trigger comparable effects on cognition.

Our literature search revealed that many studies on the effect of NIBS did not primarily study its effect on cognition, but included cognitive tests as a secondary outcome of interest. Instead, prior studies have primarily focused on utilizing neuropsychological tests as a means to observe the deterioration of cognitive abilities. As a consequence, the included tests may lack sensitivity to detect cognitive improvement, and only a few studies could be included for certain analyses which affected the power of those analyses (e.g. tDCS studies for stroke and PD; tDCS and TMS studies for TBI).

Although we aimed to avoid major differences in methodology as far as possible by careful inspection of the included studies, heterogeneity is inevitably present. The precise nature of this heterogeneity can be due to numerous factors varying across participants, data sets, studies, and brain disorders. Technical factors include the type of coil, sham technique, coil positioning, and stimulation protocol (Tables 1 and 2) (Imburgio & Orr, 2018; Lage et al., 2016; Woods et al., 2016). For example, as reflected in our retrieved studies (Tables 1 and 2) the DLPFC is one of the key anatomical regions that is most frequently targeted to improve cognition. However, we also found other (adjacent) regions to be stimulated across the included studies (e.g. Eliasova, Anderkova, Marecek, & Rektorova, 2014; Guse et al., 2013). Provided that the included studies had chosen an appropriate stimulation site based on the available literature, all stimulation sites were included in our meta-analysis.

Notably, a broad range of the cognitive tests were administered throughout the included studies. To avoid that the definition of cognitive domains would be arbitrary, we based our seven domains on the guidelines of the two most commonly used cognitive test batteries (Green et al., 2004; Litvan et al., 2012). Despite this, practice effects, ceiling and/or floor effects, and test battery sensitivity may still have contributed to heterogeneity within cognitive domains. In theory, practice effects should not play a role in randomized controlled designs, being present in both active and sham treatment groups, but with small sample sizes such effects may not cancel out. We do note that positive effects as quantified by cognitive tests may not always transfer to cognitive functioning in daily life. On the other hand, meaningful changes in cognitive functioning may not always be fully quantifiable with the cognitive tasks used. Also, post-treatment cognitive testing took place right after brain stimulation and therefore the potential long-term effect of NIBS on cognition remains understudied (Cirillo et al., 2017; Gersner, Kravetz, Feil, Pell, & Zangen, 2011).

## Conclusions

Overall, we found a small yet significant effect of both TMS and tDCS on working memory in patients with brain disorders as compared to sham treatment, tDCS also showed a superior effect on attention/vigilance. Results were not significant for the remaining cognitive domains. Findings were similar across the different brain disorders for both techniques, indicating that TMS and tDCS can only affect specific neural circuits if applied on frontal and temporal regions and hence can be applied to improve specific cognitive domains (i.e. working memory and attention/vigilance).

## Supplementary material

For supplementary material accompanying this paper visit https://doi.org/10.1017/S0033291720003670. click here to view supplementary material
