# Supplementary material for: Efficacy of non-invasive brain stimulation on cognitive functioning in brain disorders: a meta-analysis
Source: Psychol Med. 2020 Oct 19;50(15):2465–86. doi: 10.1017/S0033291720003670 (PMC7737055; doi:10.1017/S0033291720003670)
Supplement: Supplementary file 1 [file S0033291720003670sup001.zip › S0033291720003670sup013.docx]

| **Supplementary Table S2. Meta-regression for potential moderator variables.**  Slope-coefficient, according to random effects meta-regression analysis is depicted for each variable: number of sessions, age and gender. | | | | | | | |
| --- | --- | --- | --- | --- | --- | --- | --- |
| **Cognitive domain** |  | **Type** | ***n*** |  | **Nr. of sessions** | **Age** | **Gender** |
| Attention/Vigilance |  | tDCS | 923 | Slope-coefficient | -0.016 | -0.005 | -0.003 |
|  |  |  |  | *p-value* | .052 | .312 | .344 |
| Working Memory |  | TMS | 873 | Slope-coefficient | 0.001 | **0.020** | 0.001 |
|  |  |  |  | *p-value* | .941 | .005 | .883 |
|  |  | tDCS | 939 | Slope-coefficient | -0.004 | 0.001 | 0.002 |
|  |  |  |  | *p-value* | .648 | .933 | .591 |
| Note; *, *p* < .05; *n,* number of participants included in analysis; for ‘Age’, mean age of each study sample was used (if applicable), for ‘Gender’, proportion (%) females in study-sample was used (if applicable). | | | | | | | |
