# Supplementary material for: Efficacy of non-invasive brain stimulation on cognitive functioning in brain disorders: a meta-analysis
Source: Psychol Med. 2020 Oct 19;50(15):2465–86. doi: 10.1017/S0033291720003670 (PMC7737055; doi:10.1017/S0033291720003670)
Supplement: Supplementary file 1 [file S0033291720003670sup001.zip › S0033291720003670sup014.docx]

**Supplementary Table S1. Subgroup analyses for differences between disorders.** For each cognitive domain, the number of subgroups is defined by ‘k’, with degrees of freedom (k-1). A significant Q-statistic indicates significant differences in ES between disorders.

| **Cognitive domain** | **Type** | **k** | **df** | **Q-statistic** | ***p*-value** |
| --- | --- | --- | --- | --- | --- |
| **Attention/Vigilance** | TMS | 4 | 3 | 1.34 | .719 |
|  | tDCS | 7 | 6 | 4.54 | .603 |
|  | tDCS without outlier | 7 | 6 | 3.93 | .686 |
| **Working Memory** | TMS | 4 | 3 | 0.86 | .834 |
|  | tDCS | 6 | 5 | 1.42 | .923 |
| **Executive Functioning** | TMS | 6 | 5 | 5.32 | .378 |
|  | tDCS | 5 | 4 | 1.78 | .777 |
|  | tDCS without outliers | 5 | 4 | 2.90 | .575 |
| **Processing Speed** | TMS | 5 | 4 | 1.61 | .884 |
|  | tDCS | 5 | 4 | 3.68 | .452 |
|  | tDCS without outliers | 5 | 4 | **12.62** | **.013** |
| **Verbal Fluency** | TMS | 3 | 2 | 0.76 | .685 |
|  | tDCS | 3 | 2 | 0.40 | .818 |
| **Verbal Learning** | TMS | 5 | 4 | **10.76** | **.029** |
|  | TMS without outlier | 5 | 4 | 6.00 | .199 |
|  | tDCS | 5 | 4 | 2.31 | .840 |
| **Social Cognition** | tDCS | 2 | 1 | 1.73 | .19 |
| *Note:* *, *p* < .05; Bold values indicate significant differences in ES between disorders. | | | | | |
|  |  |  |  |  |  |
