# Supplementary material for: Efficacy of non-invasive brain stimulation on cognitive functioning in brain disorders: a meta-analysis
Source: Psychol Med. 2020 Oct 19;50(15):2465–86. doi: 10.1017/S0033291720003670 (PMC7737055; doi:10.1017/S0033291720003670)
Supplement: Supplementary file 1 [file S0033291720003670sup001.zip › S0033291720003670sup015.docx]

**Supplementary methods**

**Search terms**

Search terms used for each brain disorder of interest:

1. “schizophrenia”, “schizoaffective disorder”, “schizophreniform”;
2. “major depressive disorder”, “major depression”, “depression”, “MDD”, “bipolar disorder”, “bipolar depression”;
3. “Alzheimer*”, “dementia”, “mild cognitive impairment”, “MCI”, “vascular dementia”, “frontotemporal dementia”, ‘‘lewy body dementia’’, ‘‘lbd’’;
4. “parkinson*”, “Parkinson* disease”, “PD”, “lewy body dementia”, “lbd”;
5. “multiple sclerosis”, “MS”;
6. “cerebrovascular accident”, “cerebral infarct*”, “cerebrovascular infarct*”, “hemorrhagic infarct*”, “ischaemic infarct*”, “ischaemic stroke”, “cerebrovascular event”, “stroke”, “hemorrhagic stroke”;
7. “traumatic brain injury”, “TBI”

**Calculations**

Hedges’s *g* was used to quantify effect sizes (ES) for changes in cognitive performance after brain stimulation (versus sham). A positive effect size represented a superior effect of brain stimulation versus sham. Change scores were preferred over pre- and post-treatment scores to avoid overestimation of the true effect size because of the pre- and -post-treatment correlation. If not reported, pre- and post-treatment means and standard deviations (SDs), or exact F-, t- or p-values were used. For studies that did not report exact SDs, these were calculated using the 95% confidence intervals (SD=sqrt(N)*[upper limit-lower limit]/[2*1.96]) or standard error (SE) (SD=SE*sqrt(N)). When these variables were not reported, Hedges’ *g* based on change scores (end of treatment minus baseline) or exact *F*-, *p*-, *t*-, or *Z*-values were used. Studies differed largely in terms of treatment period and number of follow-up assessments. Therefore, the last follow-up time-point of active treatment of the study sample was used. Single dose (i.e., challenge) studies were included only if they had pre- and post-assessments, sensitivity analyses were run with and without these challenge studies. Studies with multiple treatment groups (for example different stimulation intensity) and one sham group were entered as individual study samples (k). When grouping studies in meta-analysis, we investigated whether studies could be combined to share a common population effect size by evaluating the *Q*-value and *I^2^–*statistic for each analysis. The *Q*-statistic tests the existence of heterogeneity, which was used to indicate the heterogeneity between the included samples. The *Q*-statistic displays a chi-square distribution with *k*-1 degrees of freedom (*k*=number of studies) and *Q*-values with *p<*.05 (two-tailed) were considered significant. The *I^2^* –statistic describes the percentage of total variation across studies due to differences in true ES rather than sampling error.

*Sensitivity analyses*

The following sensitivity analyses were performed for (trend-)significant results:

1. For studies comparing multiple interventions to the same control group, control group sample size was divided by the number of interventions included to avoid inflated ES (Borenstein *et al.,* 2009);
2. Excluding single-dose studies, to evaluate their potential influence on ES;
3. Random effects meta-regression analysis to investigate potential moderators: number of treatment sessions (including single-dose studies), mean age of study-samples and gender (%females per study sample).

Analyses described in *b.* and *c.* were performed with corrected control groups when the analysis as described in *a.* revealed relevant changes in effect size.
