# Supplementary figures and images for: Efficacy of non-invasive brain stimulation on cognitive functioning in brain disorders: a meta-analysis
Source: Psychol Med. 2020 Oct 19;50(15):2465–86. doi: 10.1017/S0033291720003670 (PMC7737055; doi:10.1017/S0033291720003670)

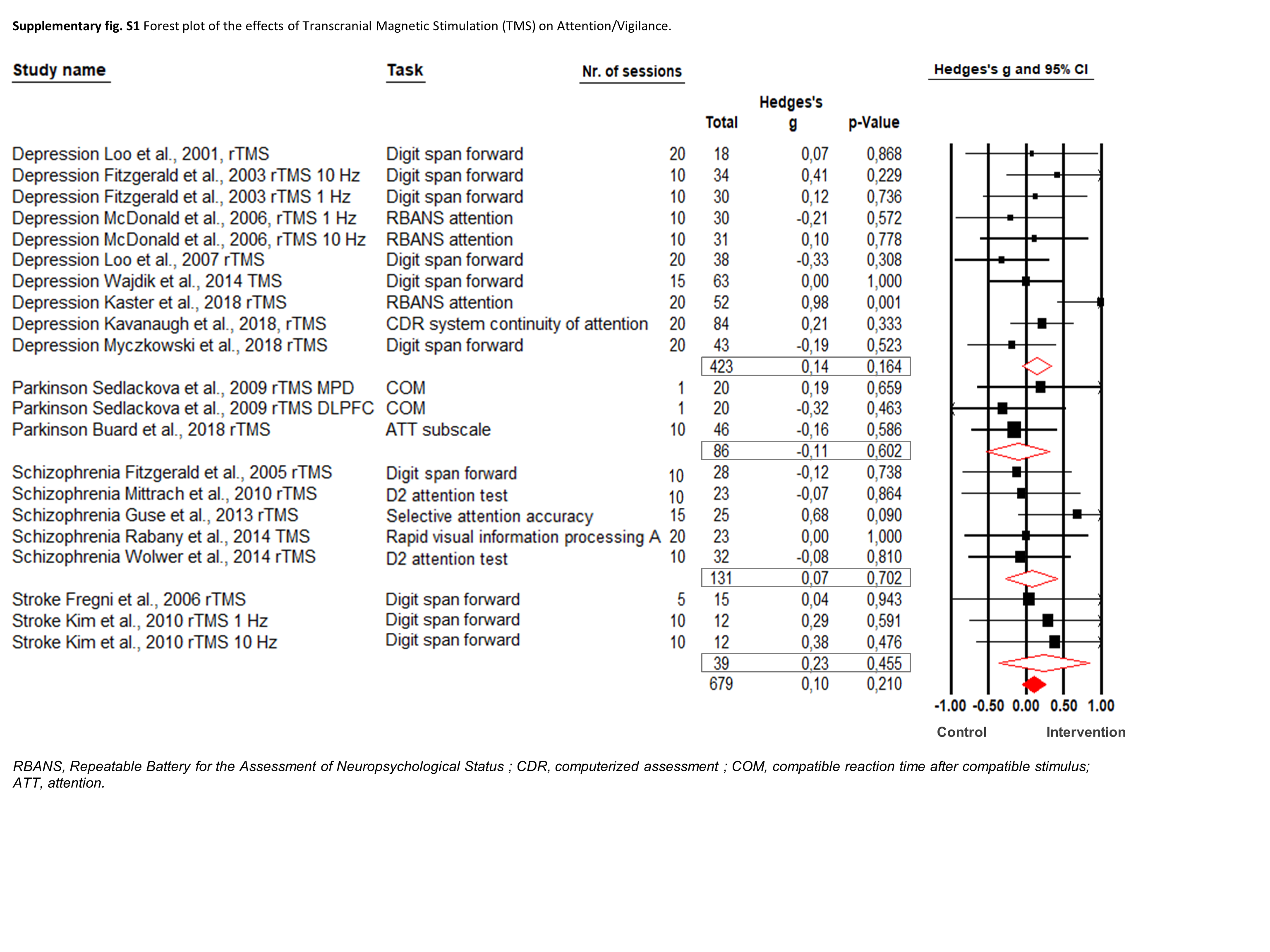

Supplement: Supplementary file 1 [file S0033291720003670sup001.zip › S0033291720003670sup001.TIF]

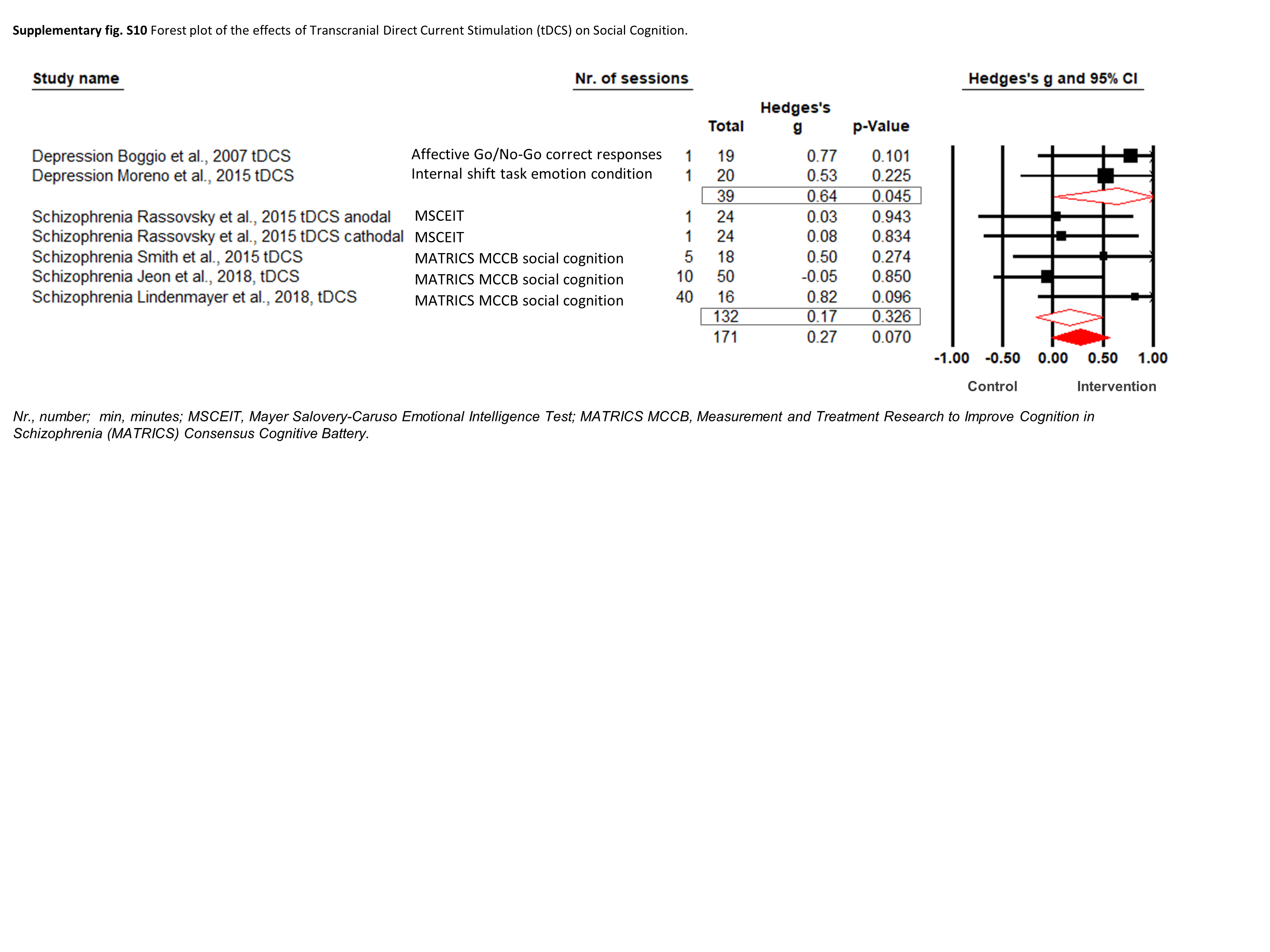

Supplement: Supplementary file 1 [file S0033291720003670sup001.zip › S0033291720003670sup002.TIF]

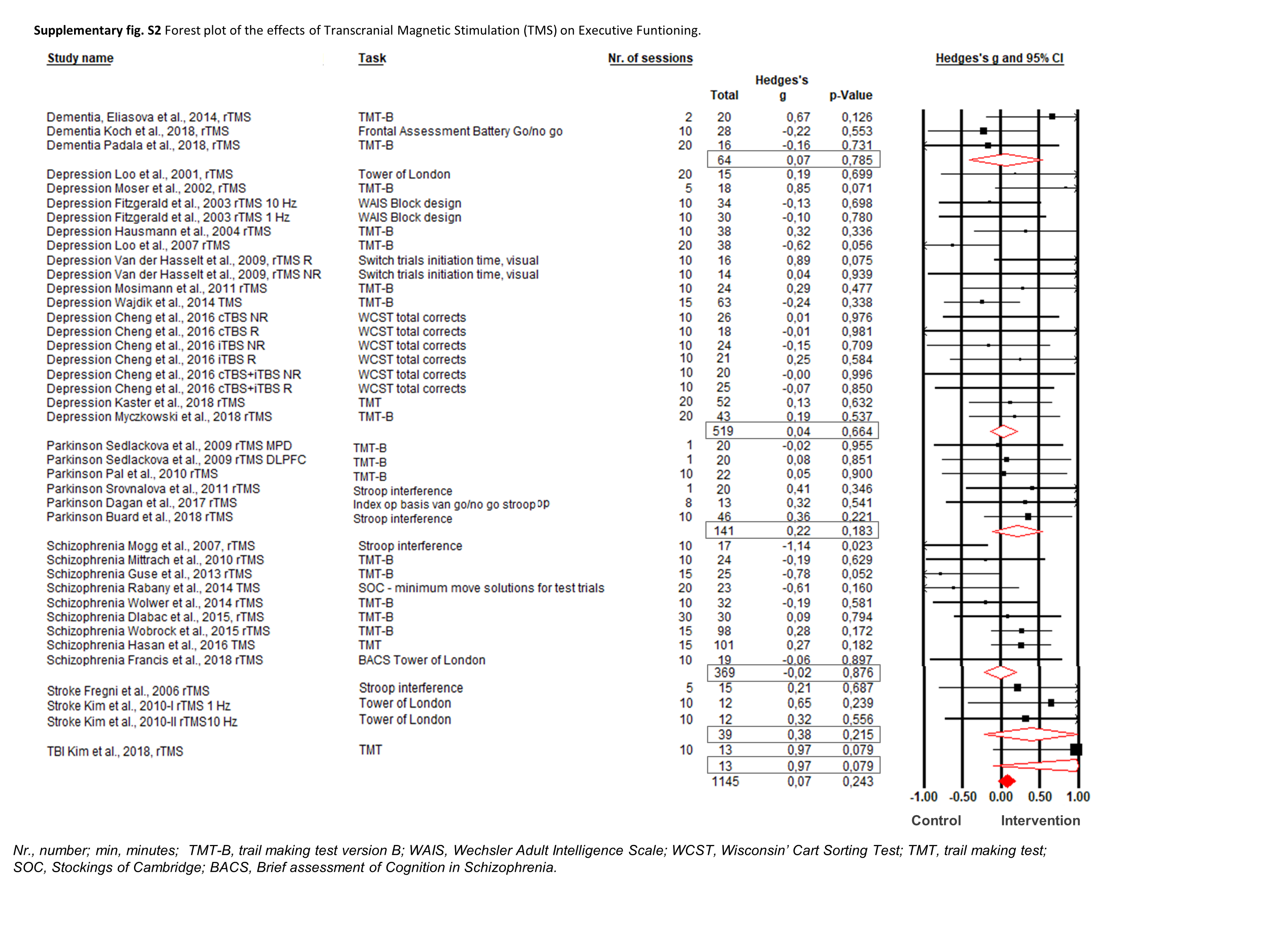

Supplement: Supplementary file 1 [file S0033291720003670sup001.zip › S0033291720003670sup003.TIF]

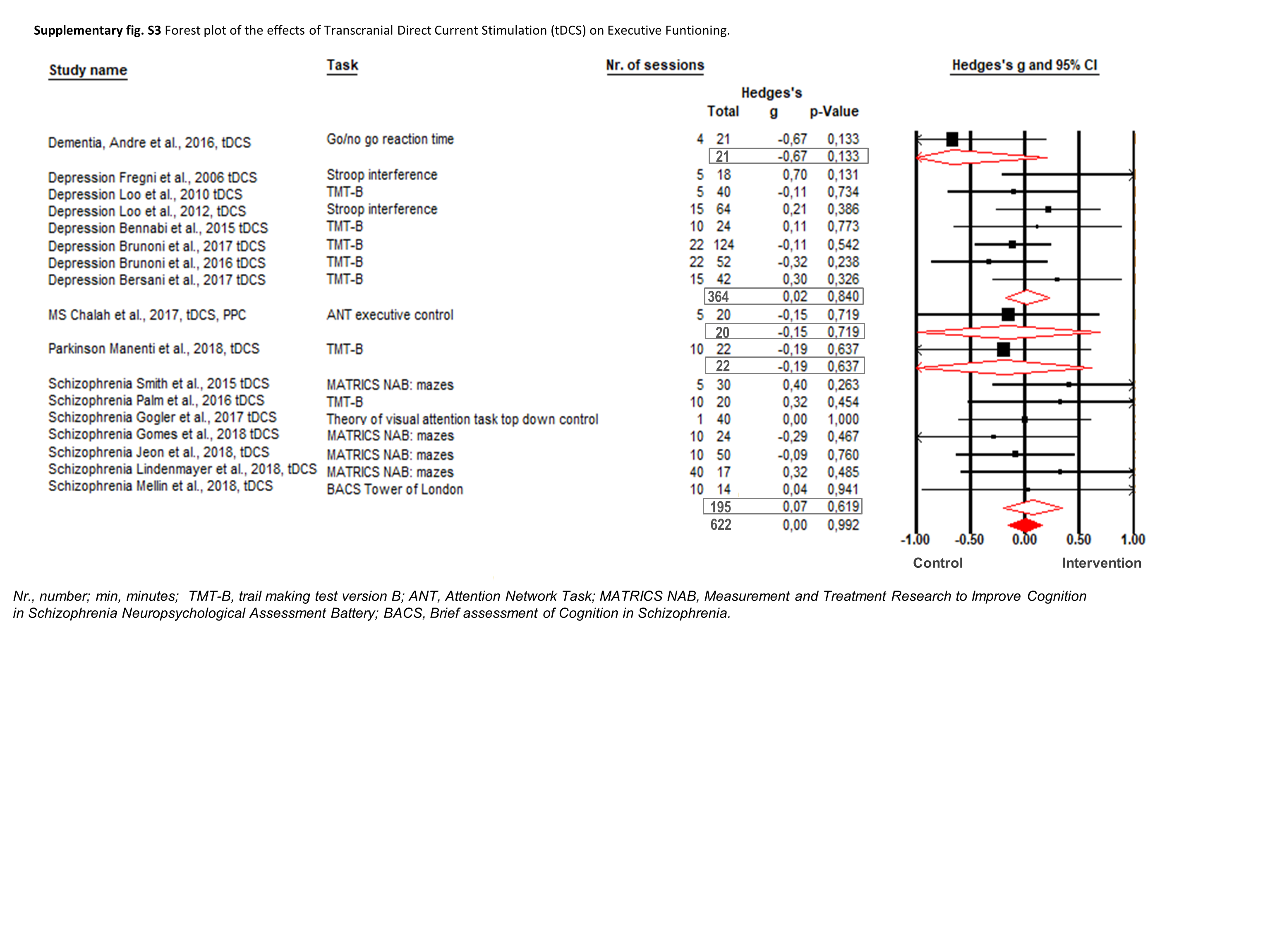

Supplement: Supplementary file 1 [file S0033291720003670sup001.zip › S0033291720003670sup004.TIF]

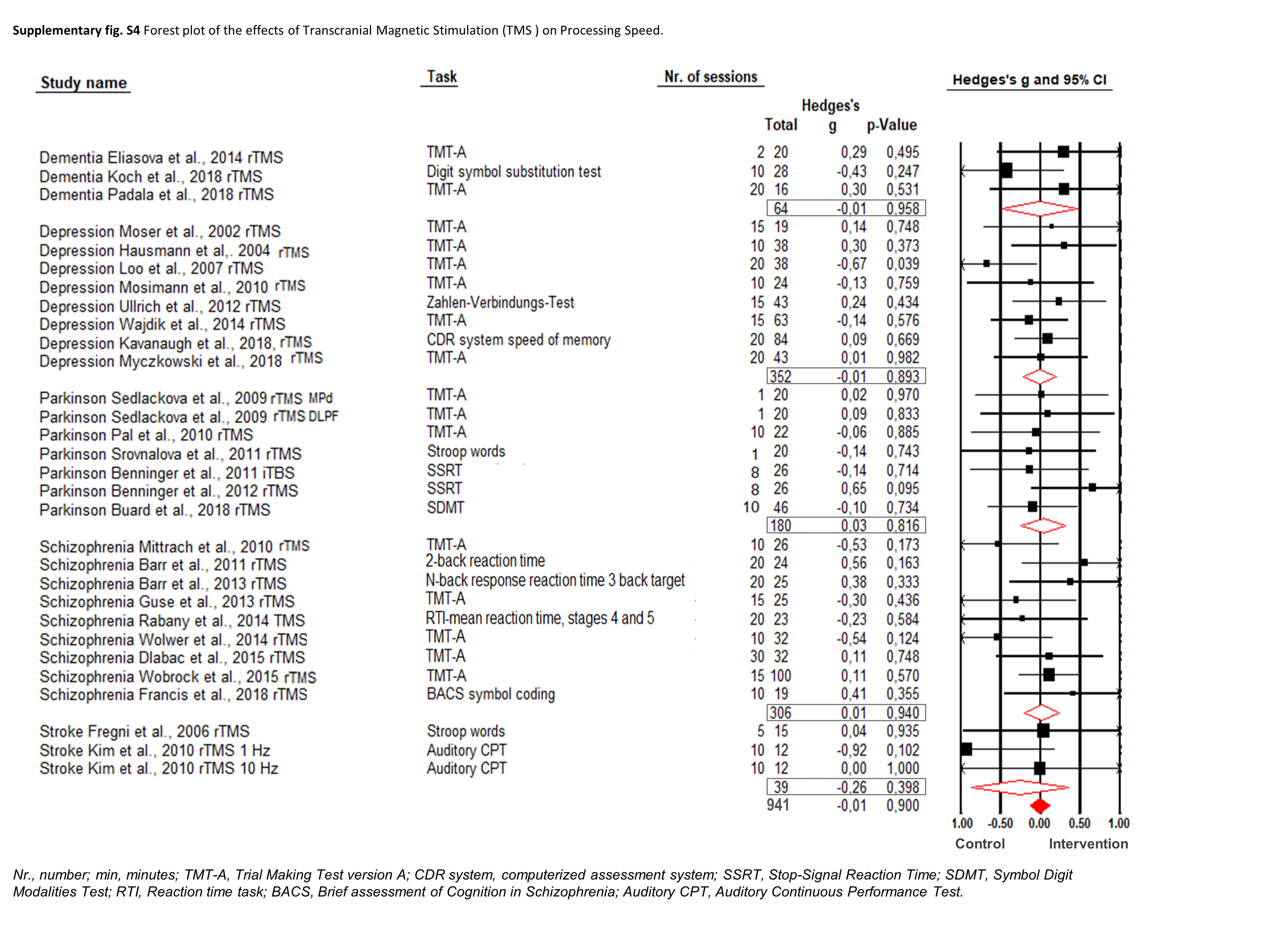

Supplement: Supplementary file 1 [file S0033291720003670sup001.zip › S0033291720003670sup005.TIF]

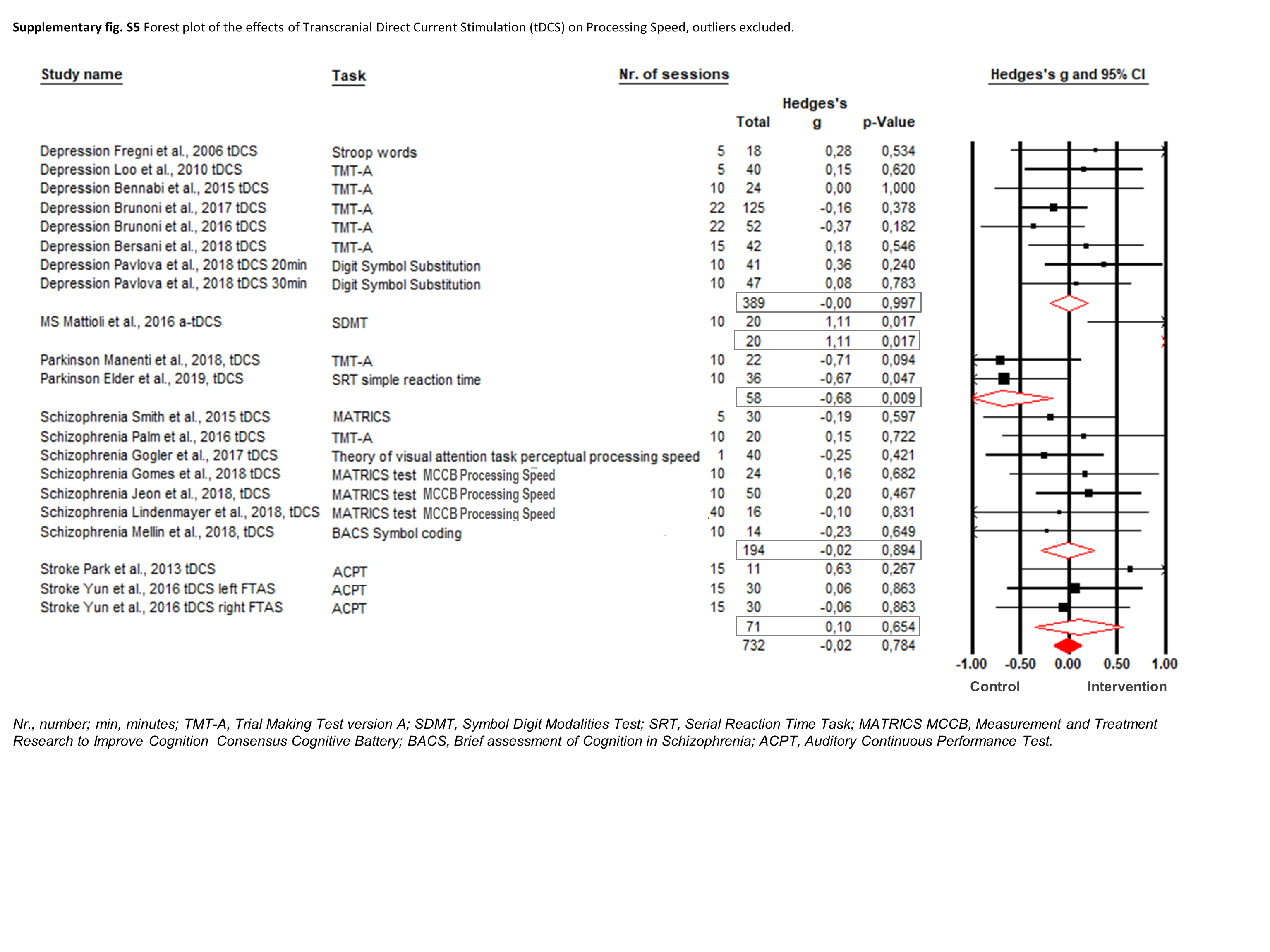

Supplement: Supplementary file 1 [file S0033291720003670sup001.zip › S0033291720003670sup006.TIF]

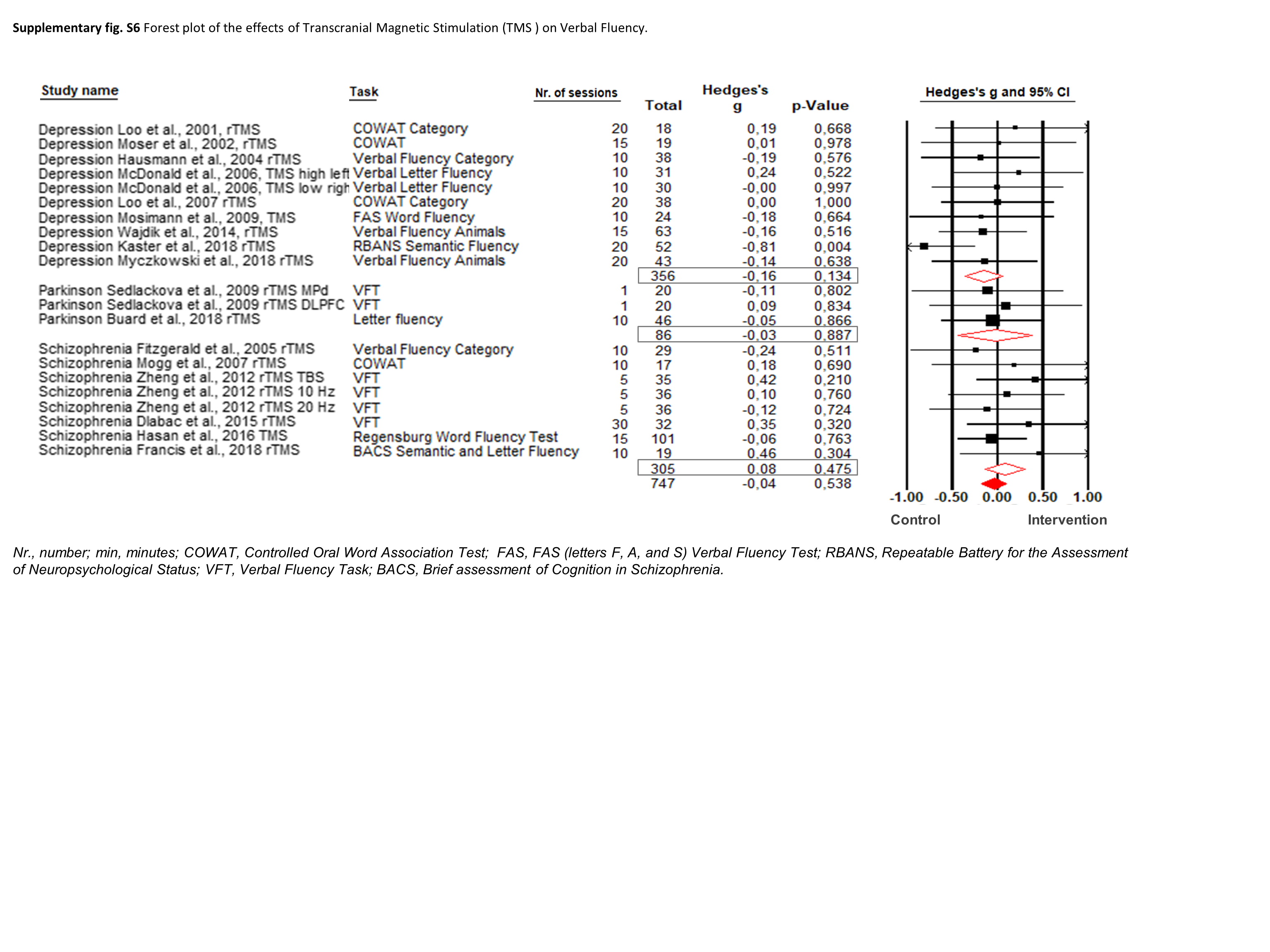

Supplement: Supplementary file 1 [file S0033291720003670sup001.zip › S0033291720003670sup007.TIF]

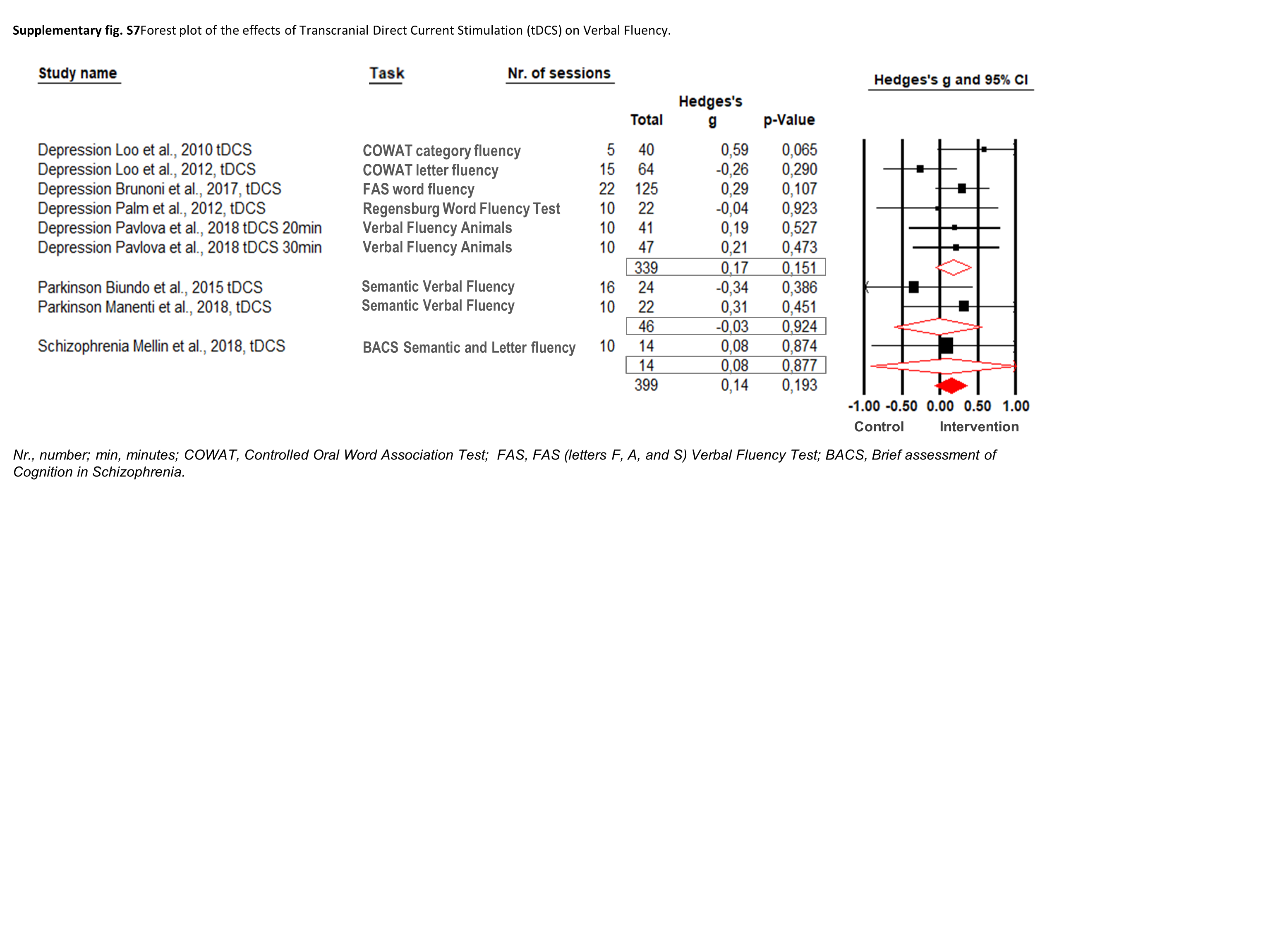

Supplement: Supplementary file 1 [file S0033291720003670sup001.zip › S0033291720003670sup008.TIF]

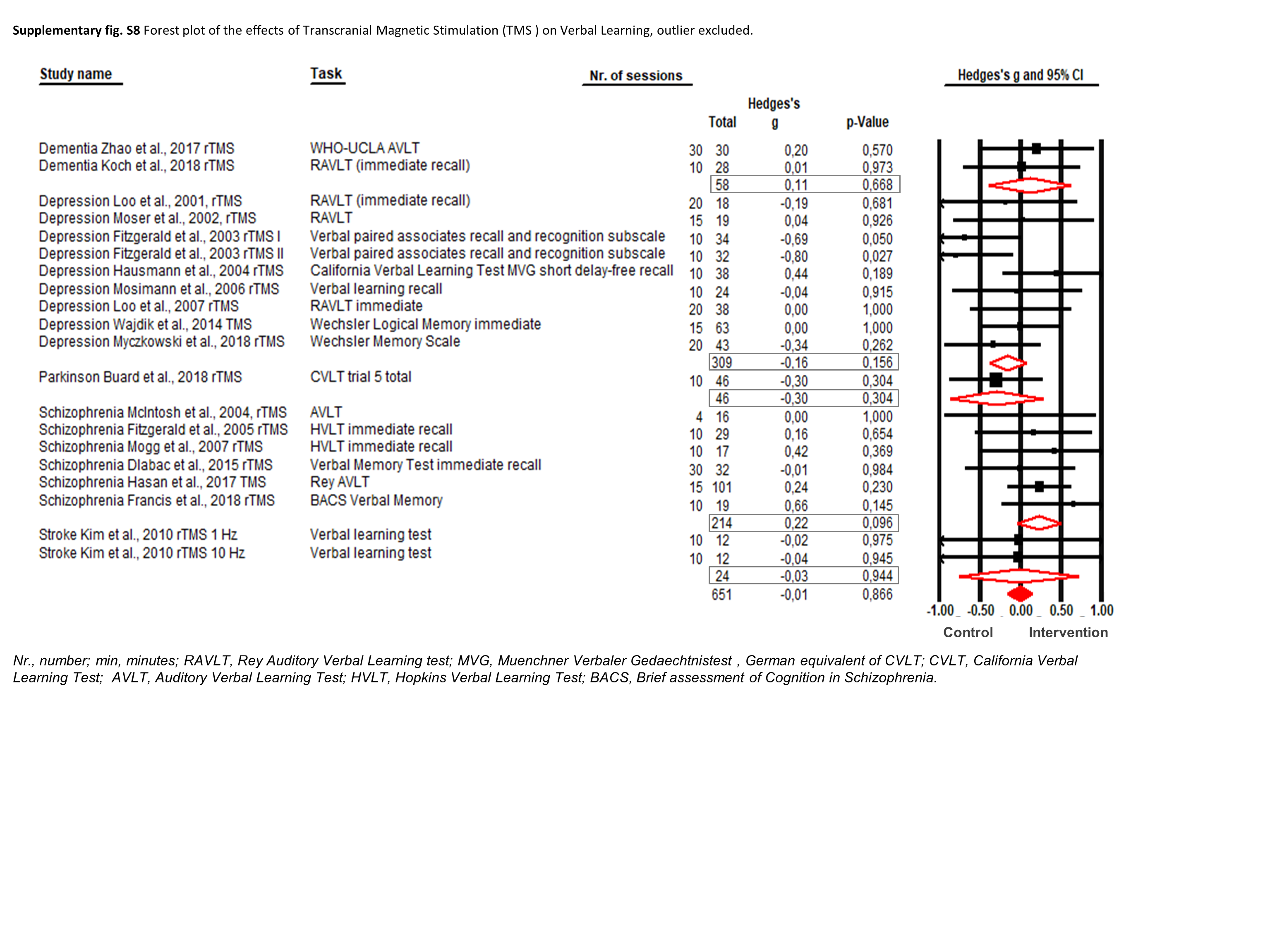

Supplement: Supplementary file 1 [file S0033291720003670sup001.zip › S0033291720003670sup009.TIF]

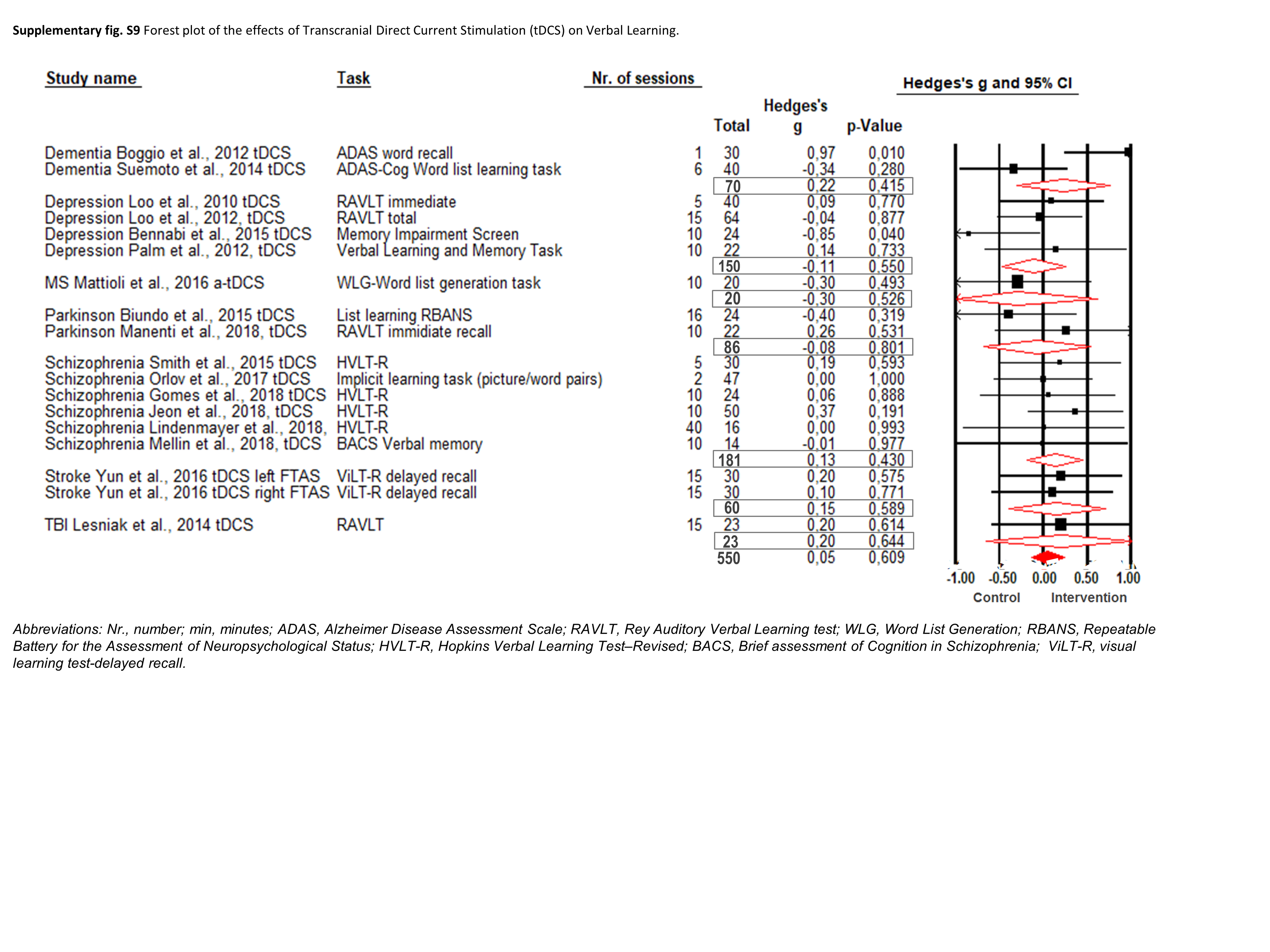

Supplement: Supplementary file 1 [file S0033291720003670sup001.zip › S0033291720003670sup010.TIF]

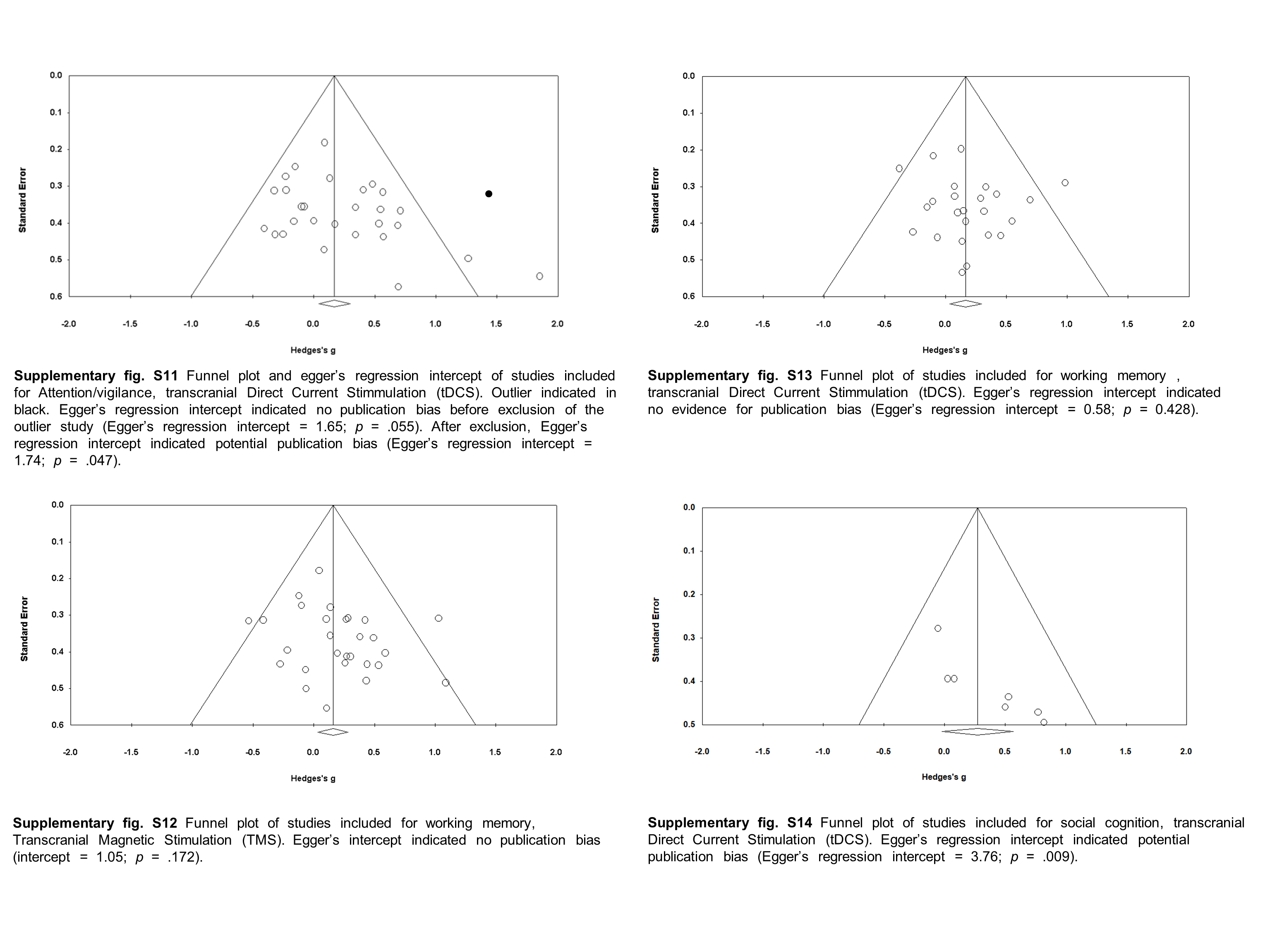

Supplement: Supplementary file 1 [file S0033291720003670sup001.zip › S0033291720003670sup012.tif]
